# Novel Quinoline- and Naphthalene-Incorporated Hydrazineylidene–Propenamide Analogues as Antidiabetic Agents: Design, Synthesis, and Computational Studies

**DOI:** 10.3390/ph17121692

**Published:** 2024-12-15

**Authors:** Osama Alharbi, Wael H. Alsaedi, Mosa Alsehli, Saif H. Althagafi, Hussam Y. Alharbi, Yazeed M. Asiri, Ramith Ramu, Mohammed Al-Ghorbani

**Affiliations:** 1 Department of Chemistry, Faculty of Science, Taibah University, Madinah 42353, Saudi Arabia; oamharbi@taibahu.edu.sa (O.A.); wsadi@taibahu.edu.sa (W.H.A.); mhsehli@taibahu.edu.sa (M.A.); 2 Department of Chemistry, Faculty of Science, Al-Baha University, P.O. Box 1988, Al Baha 65431, Saudi Arabia; salthaqafi@bu.edu.sa; 3 Department of Chemistry, Faculty of Science, Taibah University, Yanbu 46421, Saudi Arabia; hharbi@taibahu.edu.sa; 4 Department of Chemistry, College of Science, Taif University, P.O. Box 11099, Taif 21944, Saudi Arabia; y.asiree@tu.edu.sa; 5 Department of Biotechnology and Bioinformatics, JSS Academy of Higher Education and Research, Mysuru 570015, India; ramith.gowda@gmail.com; 6 Department of Chemistry, College of Education, Thamar University, Dhamar 425897, Yemen

**Keywords:** quinoline, naphthalene, α-glycosidase, α-amylase and aldose reductase enzyme inhibitions, molecular docking simulation, molecular dynamics simulation

## Abstract

**Background:** Type 2 diabetes has become a significant global health challenge. Numerous drugs have been developed to treat the condition, either as standalone therapies or in combination when glycemic control cannot be achieved with a single medication. As existing treatments often come with limitations, there is an increasing focus on creating novel therapeutic agents that offer greater efficacy and fewer side effects to better address this widespread issue. **Methods**: The methylene derivatives **3a**,**b** were coupled with phenyl/ethyl isothiocyanate in the basic medium, and dimethyl sulfate was subsequently added. Further, **5a**–**d** were reacted with the quinoline/naphthalene hydrazides **6a**,**b**. The target compounds **7a**–**g** were subjected to the in vitro enzyme inhibition studies on α-glucosidase, α-amylase, and aldose reductase. **Results**: **7g** exerted remarkable inhibitory effects on α-glycosidase [Inhibitory Concentration (IC_50_): 20.23 ± 1.10 µg/mL] and α-amylase (17.15 ± 0.30 µg/mL), outperforming acarbose (28.12 ± 0.20 µg/mL for α-glycosidase and 25.42 ± 0.10 µg/mL for α-amylase), and exhibited a strong inhibition action on aldose reductase (12.15 ± 0.24 µg/mL), surpassing quercetin (15.45 ± 0.32 µg/mL) and the other tested compounds. In a computational study, **7g** demonstrated promising binding affinities (−8.80, −8.91 kcal/mol) with α-glycosidase and α-amylase, compared to acarbose (−10.87, −10.38 kcal/mol) for α-glycosidase and α-amylase. Additionally, **7g** had strong binding with aldose reductase (−9.20 kcal/mol) in comparison to quercetin (−9.95 kcal/mol). Molecular dynamics (MDs) simulations demonstrated that **7g** remained stable over a 100 ns simulation period, and the binding free energy estimates remained consistent throughout this time. **Conclusions**: We reported the modification of quinoline and naphthalene rings to hydrazineylidene–propenamides **7a**–**g** using various synthetic approaches. **7g** emerged as a leading candidate, exhibiting greater inhibition of α-glycosidase, α-amylase, and aldose reductase. These findings underscore their potential as essential molecules for the development of innovative antidiabetic treatments.

## 1. Introduction

**Figure 1:**
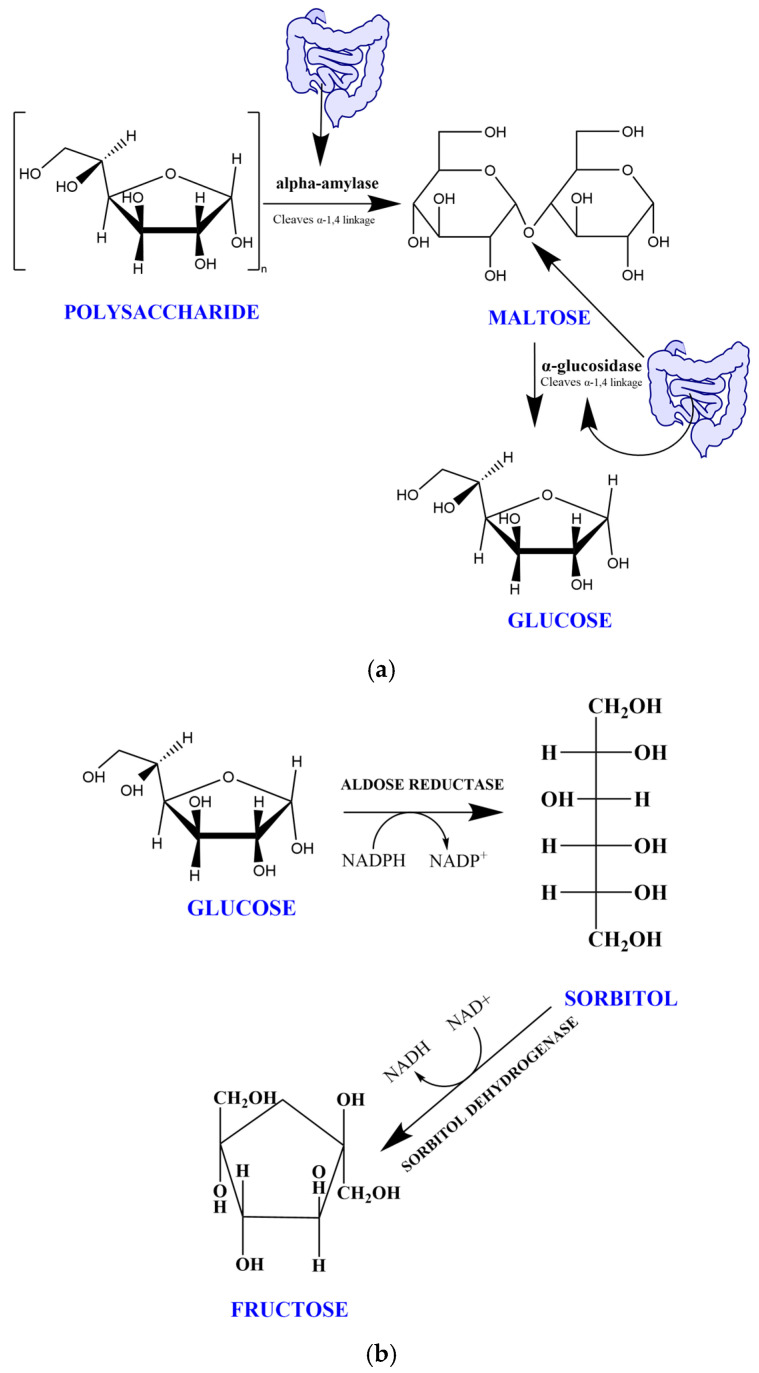
(**a**) Function of α-amylase and α-glucosidase. (**b**) Function of aldose reductase.

A chronic metabolic condition known as diabetes mellitus results from the improper functioning of insulin, a hormone produced by the pancreas that controls the metabolism of carbohydrates and lipids. This dysfunction leads to elevated blood glucose levels. The International Diabetes Federation (IDF) estimates that by 2035, the diabetic population could reach 592 million, leading to an annual treatment cost projected at USD 548 billion [1]. The rapid breakdown of dietary carbohydrates by glycoside hydrolases (such as α-glucosidase and α-amylase) converts these carbohydrates into monosaccharides, resulting in increased blood glucose levels, a condition known as postprandial hyperglycemia. This condition serves as an early indicator of diabetes, and the use of glycosidase inhibitors, such as acarbose and miglitol, is a well-established method for managing postprandial hyperglycemia. These inhibitors function by obstructing the release of glucose units, thereby promoting more stable glucose levels [2,3]. By slowing the activity of α-amylase (inhibitors acarbose and voglibose) and α-glucosidase, which are responsible for breaking down complex carbohydrates in the small intestine, these inhibitors delay carbohydrate absorption and reduce the rise in post-meal glucose levels [4,5] (Figure 1a,b). On the other hand, amide linkers are of significant importance across various fields, including chemistry, pharmaceuticals, and bioengineering. They play pivotal roles in chemical synthesis, drug development, and polymer science [6,7,8].

**Figure 2:**
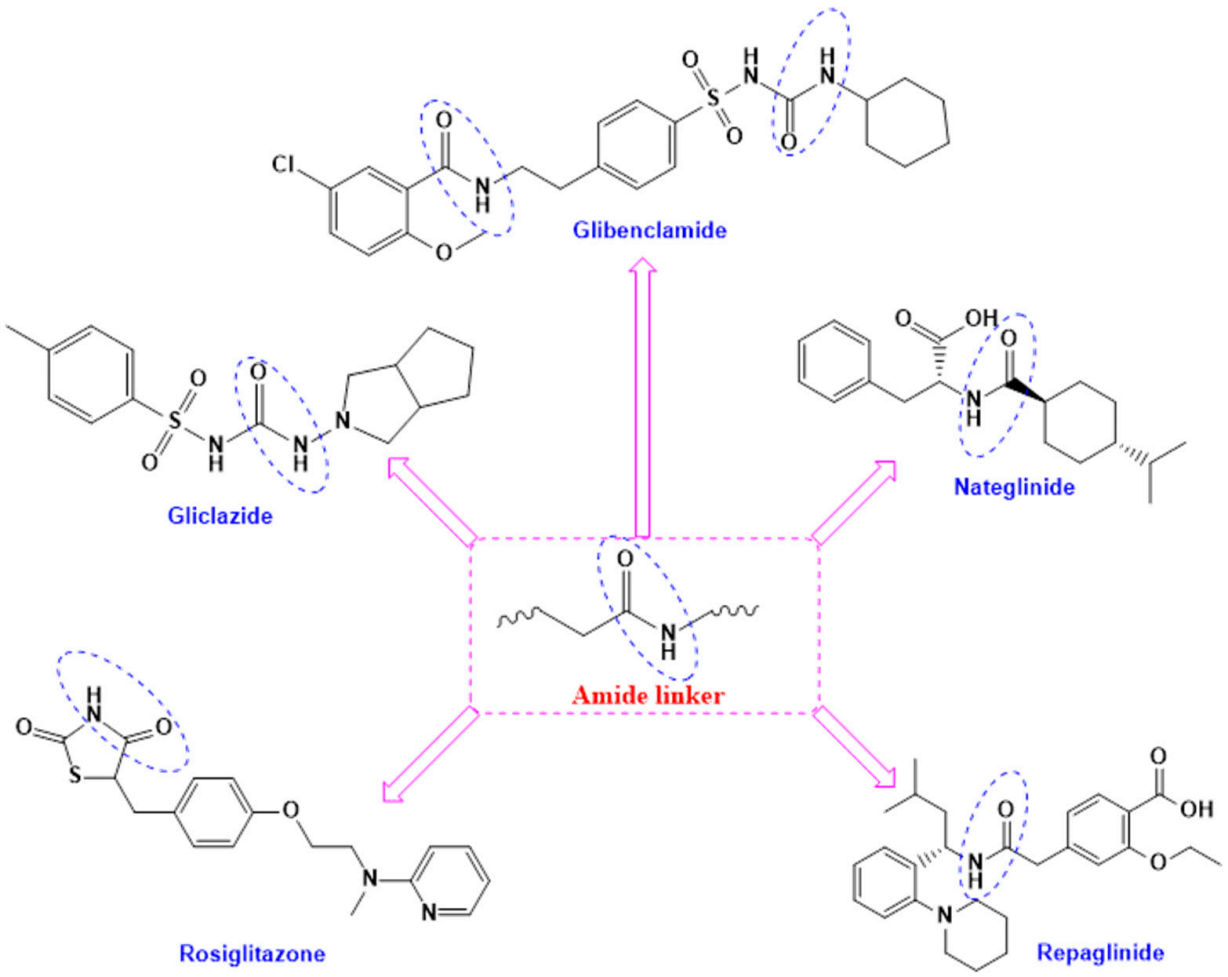
Fundamental pharmacophoric features of the amide linker in antidiabetic drugs.

Bioisosterism serves as a strategic tool for the systematic modification of lead compounds, with the aim of enhancing their potency, selectivity, and pharmacokinetic profiles, while also mitigating toxicity and expanding intellectual property through the exploration of novel compounds. The amide group is also a crucial component of biomolecules, playing a pivotal role in the structure and function of several approved medications for type 2 diabetes, such as Glibenclamide, Nateglinide, Gliclazide, Repaglinide, and Rosiglitazone. These drugs have been utilized as antidiabetic medications, underscoring the significant role of this functional group in their therapeutic efficacy (Figure 2). Quinoline and naphthalene have emerged as vital fused-cyclic compounds that serve as fundamental frameworks for drug discovery and hold significant importance in various biological and pharmaceutical applications [9,10,11]. Numerous studies demonstrating both natural products and synthetic derivatives featuring a quinoline framework have shown a variety of beneficial bioactivities, including antibacterial [12,13], antiviral [14], and antioxidant activity [15]. Previously, our research team synthesized quinoline derivatives featuring an amide linkage, exploring their potential for antibacterial, antifungal, and antidiabetic applications [16,17]. In the present study, we investigated the antidiabetic properties of quinoline and naphthalene derivatives through in silico and in vitro enzyme inhibition studies.

### Rationale and Structure-Based Design

**Figure 3:**
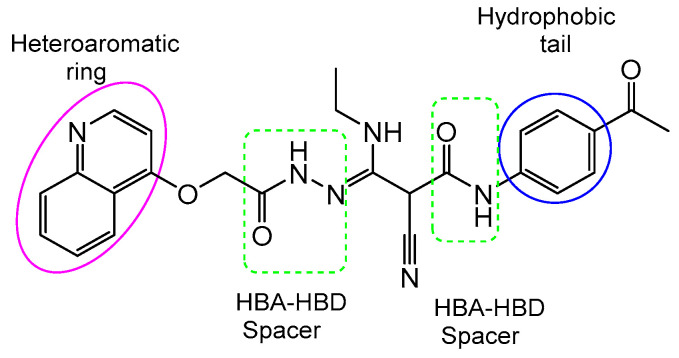
Design of the target compound **7g**.

The target derivatives were designed by leveraging the bioisosteric features of amide-based type 2 antidiabetic drugs (Figure 1). This involved synthesizing a series of amide-fused rings. Four components make up the ATP binding domain: a fused heteroaromatic ring, a hydrogen bond accepter and donor, a linker, and a terminal hydrophobic moiety that fits into the allosteric hydrophobic pocket (Figure 3). Our intention was to attach large, fused rings of naphthalene and quinoline to a newly formed pyrazole core to improve their antidiabetic activity. The selective formation of the amide linkage, as opposed to alternative ring-closure reactions such as pyrazole formation, is attributed to the steric hindrance imparted by the phenyl and ethyl groups on the NH group. Hence, amide was hybridized with quinoline and naphthalene to create potentially more-effective candidates. A series of new compounds was developed based on the quinoline–amide scaffold. The antidiabetic activity of these compounds was investigated. The hybrid group showed promising activity, while modifications were focused on linking the heteroaromatic ring (quinoline) with acetophenone through the amide linker to form a hydrazone moiety **7g**.

## 2. Results and Discussion

### 2.1. Chemistry

**Scheme 1:**
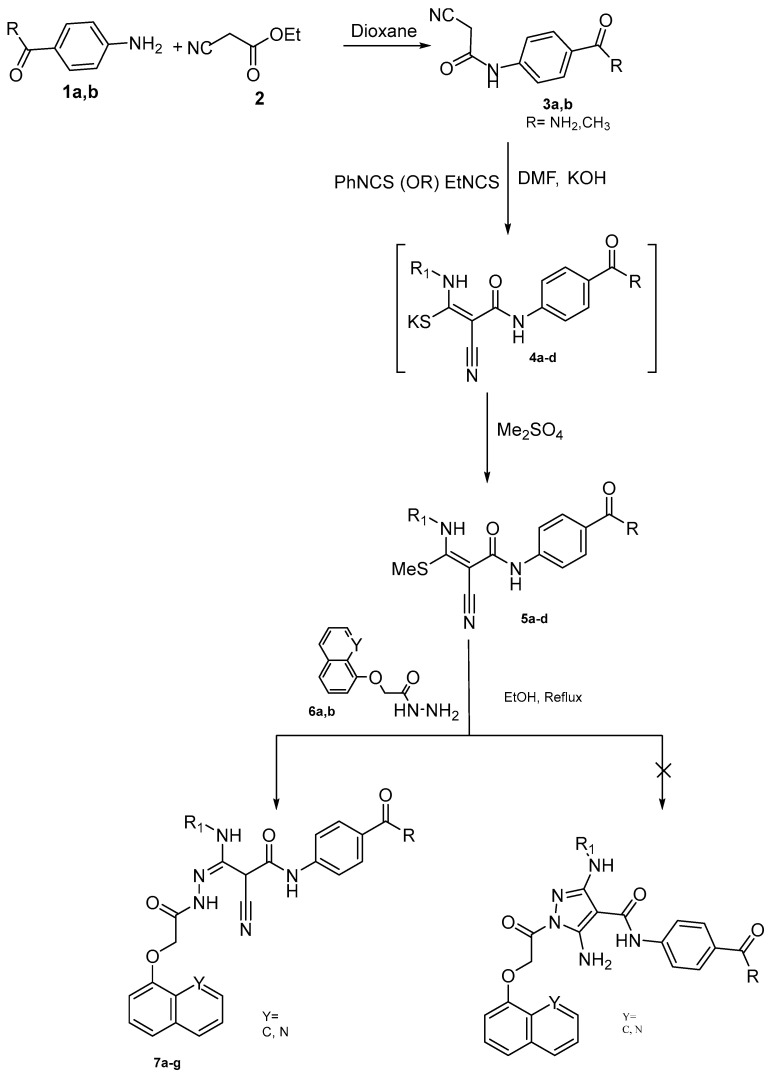
Synthesis of quinoline and naphthalene derivatives **7a**–**g**.

Substituted (quinolin-4-yloxy/naphthalen-2-yloxy) acetyl hydrazineylidene propenamides (**7a**–**g**) were synthesized using the synthetic method outlined in Scheme 1. Our previous studies demonstrated the successful reaction of phenyl isothiocyanate with active methylene compounds under basic conditions, providing an effective route for producing thiazoline, thiazolidine, and thiophene ring structures [8]. Continuing with this research, we have extended our synthetic efforts to produce new derivatives utilizing N-(4-acetylphenyl)-2-cyanoacetamide and 4-(2-cyanoacetamido) benzamide (**3a**,**b**), PhNCS, and EtNCS as key starting materials. It is well established that a diverse array of reactants containing the N–C–S motif readily undergo coupling upon reaction. We present an extension of this synthetic approach by treating basic methylene derivatives, namely, N-(4-acetylphenyl)-2-cyanoacetamide and 4-(2-cyanoacetamido) benzamide (**3a**,**b**), with phenyl/ethyl isothiocyanate in DMF at room temperature. Furthermore, the addition of dimethyl sulfate to thiocarbamoyl analogues **4a**–**d** yielded ketene, N, S, and acetal **5a**–**d**. These intermediates, **5a**–**d**, offer a versatile basis for the synthesis of various derivatives. Consequently, the coupling reaction of **5a**–**d** with the hydrazide analogues **6a**,**b** produced compounds **7a**–**g**. From the analysis data, we observed that the coupling reaction was more specific than the formation of the pyrazole ring through cyclization, which can be ascribed to the steric hindrance caused by the phenyl and ethyl groups on NH. The target compounds **7a**–**g** were recognized by Infrared (IR) and Nuclear Magnetic Resonance (NMR) spectroscopy. The IR of compounds **7a**–**g** revealed absorption bands spanning 3134–3489, 2191–2202, 1652–1682, and 1608–1626, which correspond to the NH_2_, NH, CN, C=O, and C=N groups, respectively. The NMR spectrum of **7g**, which is an instance of the prepared series, shows five singlet signals at δ 10.54, 9.35, 7.48, 4.86, 2.64, and 1.20 ppm due to 2NH, NH_2_, CH_2_, CH_3_, and qurtite, as well as triplet signals at δ 3.57 and 1.20 ppm due to CH_2_ and CH_3_, respectively. Furthermore, ^13^C NMR revealed signals at 170.55, 166.24, 138.19, 75.09, 18.09, and 15.81, which are associated with the C=O, CN, CH_2_, and 2CH_3_ carbons.

### 2.2. Biology

#### In Vitro Carbohydrate-Digesting Enzymes Inhibition Assays

The in vitro enzyme inhibition studies on α-glucosidase, α-amylase, and aldose reductase revealed that compound **7g** demonstrated the most effective inhibitory potential among all the compounds, **7a**–**g**. For α-glucosidase, **7g** had an Inhibitory Concentration (IC_50_) value of 20.23 ± 1.10 µg/mL, outperforming acarbose (IC_50_: 28.12 ± 0.20 µg/mL). Similarly, **7g** exhibited the strongest α-amylase inhibition (IC_50_: 17.15 ± 0.10 µg/mL), surpassing acarbose (IC_50_: 25.42 ± 0.10 µg/mL). For aldose reductase, **7g** also showed superior inhibition (IC_50_: 12.15 ± 0.24 µg/mL) compared to quercetin (IC_50_: 15.45 ± 0.32 µg/mL). According to the results, compound **7g** clearly exhibited a severe inhibition of all the enzymes, based on its IC_50_ values, which were significantly higher (*p* < 0.05) than those of acarbose and quercetin (Table 1). The dose-dependent inhibition data (% inhibition) of compound **7g** is provided in the Supplementary Materials, Table S1. The results of the in vitro assays for the inhibition of carbohydrate-digesting enzymes were matched with the Al-Ghorbani et al. study, which found that the pyridine derivative inhibited the two enzymes that hydrolyze carbohydrates [16].

### 2.3. Computational Assays

#### 2.3.1. Molecular Docking Studies

**Figure 4:**
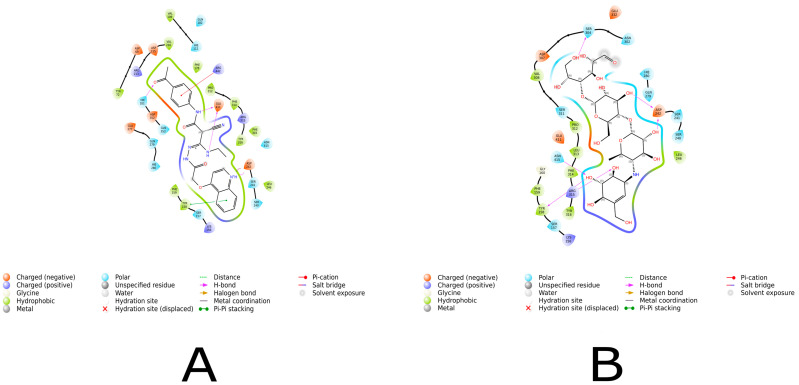
Compound **7g** interacting with active site amino acids of α-glucosidase are represented in two dimensions (**A**), and acarbose interacting with the active site amino acids of α-glucosidase are represented in two dimensions (**B**).

Three antidiabetic targets, namely α-glucosidase, α-amylase, and aldose reductase, were docked with derivatives **7a**–**g** and two positive controls, acarbose and quercetin. Compound **7g** inhibited all three carbohydrate-digesting enzymes that had the highest glide score in Table 2. With respect to α-glucosidase, compound **7g** interacted with the glide score of −8.80 kcal/mol by forming three hydrogen bonds with His 351 and Glu 411. Other interactions included the Pi -Pi stacking interactions with Tyr 158, the Pi-cation interaction with Arg 442, and the salt bridge with Asp 242. Conversely, the standard inhibitor, acarbose, interacted with the glide score of −10.87 kcal/mol by forming six H-bonds with Asp 242, Asn 415, Tyr 158, Arg 315, and Ser 304. The 2D representations of α-glucosidase–compound **7g** and α-glucosidase–acarbose are depicted in Figure 4.

**Figure 5:**
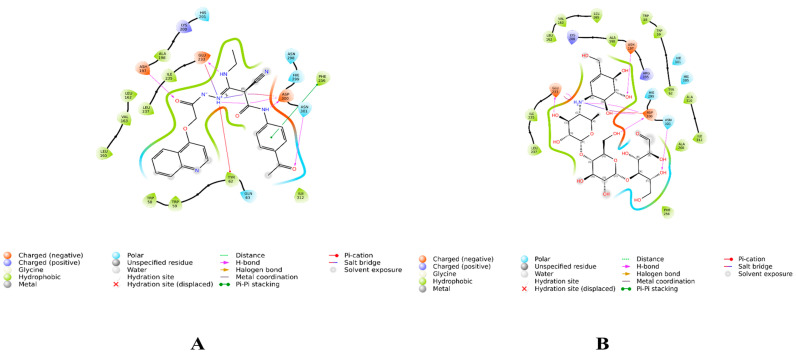
Compound **7g** interacting with active site amino acids of α-amylase are represented in two dimensions (**A**), and acarbose interacting with the active site amino acids of α-amylase are represented in two dimensions (**B**).

Regarding α-amylase, **7g** interacted with the enzyme with a glide score of −8.91 kcal/mol, creating three hydrogen bonds with its catalytic site residues (Asp 197, Glu 233, and Asp 300), which resulted in the enzyme’s total inhibition. Additional interactions included the Pi-Phe 256 stacking interaction, the salt bridge with Asp 300, the Glu 233 interaction, and the Pi-cation interaction with Tyr 62. As an alternative, the typical inhibitor, acarbose, formed four hydrogen bonds with Asp 197, Glu 233, Asp 300, and Asn 301 to interact with the glide score of −10.38 kcal/mol. Other interactions were noted, like the salt bridge with Glu 233 and Asp 300. Figure 5 shows the 2D representations of α-amylase–compound **7g** and α-amylase–acarbose.

**Figure 6:**
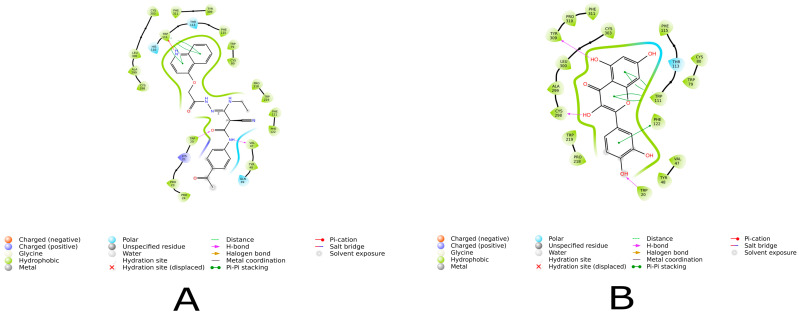
Compound **7g** interacting with active site amino acids of aldose reductase are represented in two dimensions (**A**), and acarbose interacting with the active site amino acids of aldose reductase are represented in two dimensions (**B**).

In addition, compound **7g** shaped two hydrogen bonds with Trp 20 and Val 47 to interact with aldose reductase, achieving a glide score of −9.20. Additional interactions with Trp 111 were discovered, including Pi-P stacking and Pi-cation interactions. Conversely, though, the positive control quercetin formed three hydrogen bonds with Trp 20, Cys 298, and Tyr 309 in response to the glide score of −9.95 kcal/mol. Additional interactions were also noted, including Pi-Pi stacking with Trp 111 and Phe 122. According to the results above, compound **7g** had the same glide score and interactions as the positive controls, despite the positive controls having higher glide scores and interactions. Figure 6 shows the 2D representations of aldose reductase–**7g** and aldose reductase–acarbose.

**Figure 7:**
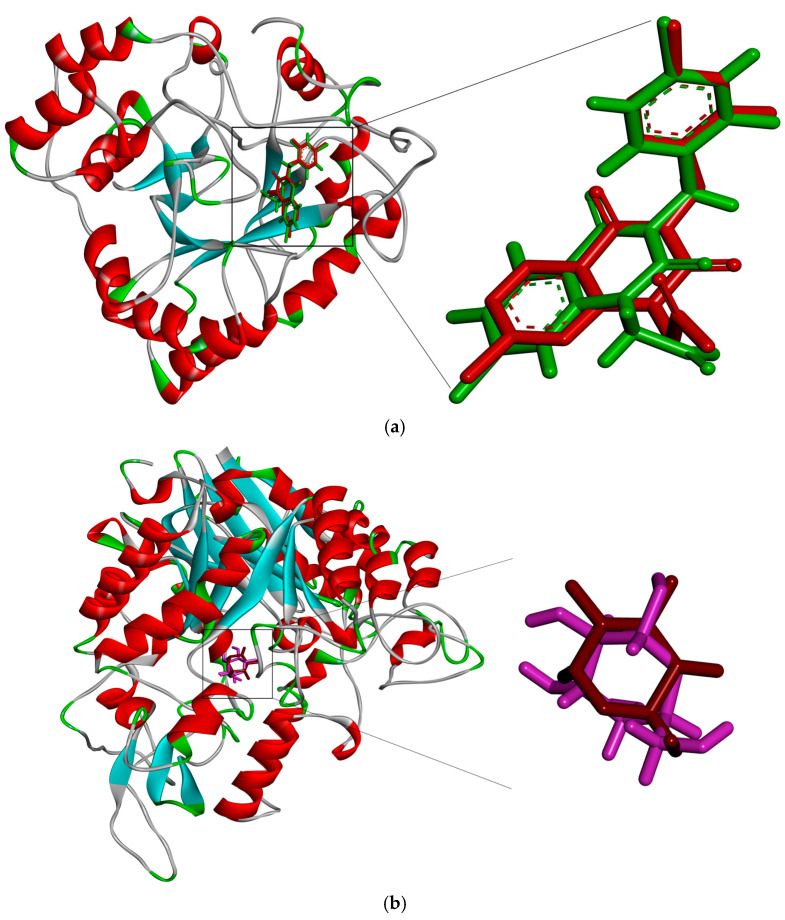
(**a**) The validation of molecular docking by the superimposition of the original and re-docked structures of the co-crystal ligand Zenerastat. Green: original compound from PDB. Red: re-docked compound. (**b**) The validation of molecular docking by the superimposition of the original and re-docked structures of the co-crystal ligand GLC 601. Pink: original compound from PDB. Brown: re-docked compound.

The molecular docking analysis results were reliable with the previous study [18,19], which found that caffeic acid, extracted from whole jackfruit flour, bound to the same amino acid involved in catalytic activity, thereby inhibiting the three antidiabetic targets. But compared to compound **7g**, caffeic acid showed low binding affinities (−8.2, −8.1, and −7.4 kcal/mol against α-glucosidase, α-amylase, and aldose reductase, respectively) and more hydrogen bonds than **7g**. The polyphenol rutin was also found in whole jackfruit flour bound to the same amino acids involved in the catalytic activity, inhibiting the three antidiabetic targets. The molecular docking results corroborated these findings. During the validation of the molecular docking simulation, the RMSD between the original and re-docked co-crystal ligands GLC 601 and Zenerastat was found to be 0.000, which proved the docking protocol was right (Figure 7a,b).

#### 2.3.2. Molecular Dynamics Simulation

##### Simulation of Molecular Dynamics of α-Glucosidase with Compound **7g** and Acarbose

The simulations examine the dynamic interactions between protein–ligand complexes within a solvated environment (Cui et al., 2008) [20]. These studies evaluate various aspects of the protein–ligand complex, including its root mean square deviation (RMSD), radius of gyration (Rg), and solvent-accessible surface area (SASA). Additionally, they assess the ligand RMSD, hydrogen bonding, and changes in the secondary-structure pattern between the protein and its complexes [21]. This study was utilized to describe local variations within the protein chain. Peaks on the root mean square fluctuations (RMSF) (Figure 7) represent regions of the protein that see the greatest fluctuations throughout the simulation. The protein’s N- and C-terminal tails often exhibit greater fluctuations than any other region. Secondary structural elements, such as alpha helices and beta strands, tend to change less than loop sections due to the fact that they are more rigid than the formless portions of the protein. The ligand RMSD (right Y-axis) demonstrates the ligand stability concerning the protein and its binding pocket. The graph displays “Lig fit Prot”, which indicates the RMSD of the ligand following the alignment of the protein–ligand complex with the reference protein backbone. This measurement is obtained by calculating the RMSD of the ligand’s heavy atoms after the alignment process. If the values observed are significantly higher than the protein’s RMSD, it may indicate that the ligand is prevalent away from its original binding site. The RMSD of a ligand relative to the reference conformation (usually, time t = 0 is defined as the initial frame, which is utilized as the reference). Gyration radius (rGyr): equipped with its fundamental moment of inertia, it quantifies the “extendedness” of a ligand. Intra HB is the number of internal H-bonds in a ligand. MolSA: the calculation of molecular surface with the 1.4 Å probe radius. SASA: the accessible surface area of a ligand by water. PSA: the surface area of a ligand accessible to solvents that is only provided by O and N atoms.

**Figure 8:**
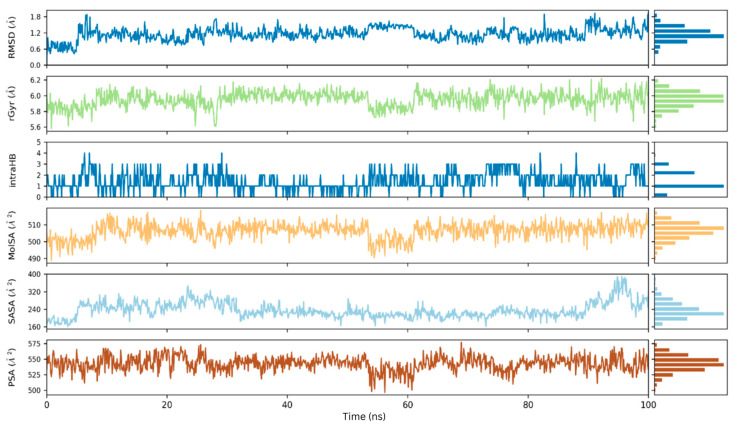
Trajectories depicting the different properties—RMSD (Å), the radius of gyration, intra hydrogen bonds, molecular surface area, solvent accessible surface area, and the polar surface area of compound **7g** when complexed with α-glucosidase.

The findings regarding the ligand properties, as derived from the MD simulation analysis displayed in Figure 8, are consistent with the research conducted by Bhaumik et al. (2024) [22]. The RMSD demonstrates a range of 0.6 Å to 1.8 Å, indicating structural alterations and a degree of stability. The histogram reveals a peak around 1.2 Å, which suggests that the ligand remains relatively stable within the active site of the target, exhibiting only minor deviations. The radius of gyration, which measures the compactness of the molecular structure, remains relatively stable at approximately 5.8 Å throughout the simulation. This constancy indicates that the ligand maintains a stable configuration, a conclusion that is further supported by the narrow distribution evident in the histogram. Furthermore, the number of intramolecular hydrogen bonds fluctuates between one and five, reflecting dynamic hydrogen bonding interactions. The broad distribution observed in this histogram implies flexibility within the hydrogen bond network. The MoISA oscillates around 500 Å^2^, with slight variations indicating changes in the exposure of surface residues. The histogram exhibits a peak at approximately 505 Å^2^, suggesting that this is the most prevalent state of surface exposure. Additionally, the SASA demonstrates an increasing trend as the simulation progresses, indicating enhanced solvent exposure over time. The histogram indicates variability, with a peak near 250 Å^2^, reflecting dynamic modifications in solvent exposure. The PSA shows fluctuations between 525 Å^2^ and 575 Å^2^, reflecting alterations in the exposure of polar residues; the histogram peak at around 540 Å^2^ indicates a predominant state of polar surface exposure. Collectively, these results indicate that the ligand remained stable within the binding site of α-glucosidase throughout the simulation.

**Figure 9:**
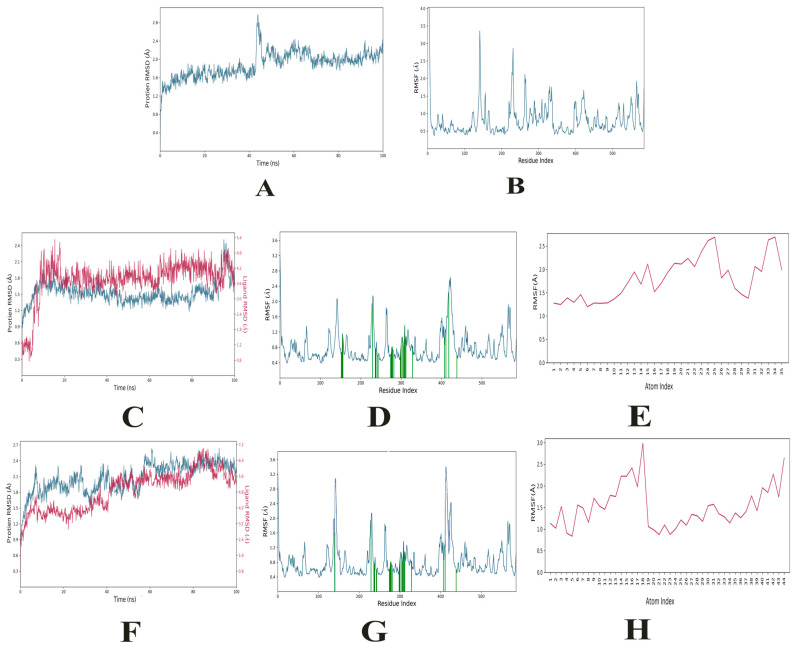
MD trajectories of α-glucosidase complexed with compound **7g** and acarbose. (**A**) RMSD of α-glucosidase. (**B**) RMSF of α-glucosidase. (**C**) RMSD of α-glucosidase–compound **7g**. (**D**) RMSF of α-glucosidase. (**E**) RMSF of compound **7g**. (**F**) RMSD of α-glucosidase–acarbose. (**G**) RMSF of α-glucosidase. (**H**) RMSF of acarbose. The pink color in Figures (**C**,**F**) indicates the RMSD of compound **7g** and acarbose, and the blue color indicates the protein (α-glucosidase) RMSD. The blue color in Figures (**B**,**D**,**G**) indicates the RMSF of the protein i.e., in this case, it is α-glucosidase. The pink color in Figures (**E**,**H**) indicates the RMSF of the compound **7g** and acarbose respectively. The green color in Figures (**D**,**G**) indicates Proteins (amino acid residues) that interact with the ligand, which are marked with green-colored vertical bars.

The RMSD of α-glucosidase was analyzed over a 100 ns MD simulation to assess the stability of the protein–ligand complex (Figure 9). The results align with the study by Askarzadeh et al. (2022) [23], which indicated that the RMSD of the unbound enzyme significantly increased during the first 7.5 ns, reaching a value of 2.5 Å. After this initial spike, the RMSD exhibited steady fluctuations over the next 30 ns before stabilizing during the final 15 ns of the simulation at approximately 2.6 Å. In this study, the RMSD values of the α-glucosidase–acarbose and α-glucosidase–compound **7g** complexes showed that these enzyme–ligand complexes-maintained stability throughout the simulation. The RMSD value for the apoprotein peaked at 2.4 Å. In comparison, the RMSD of the α-glucosidase–compound **7g** complex demonstrated an RMSD of 0.9 Å (for apoprotein) and 3.6 Å (for the compound **7g**–α-glucosidase complex), and the α-glucosidase–acarbose complex had an RMSD of 1.2 Å (apoprotein) and 5.6 Å (for α-glucosidase–acarbose).

Regarding RMSF, compound **7g** exhibited the highest fluctuation, reaching 2.5 Å. The α-glucosidase enzyme interacted with key residues, including His 351, Glu 411, Tyr 158, Arg 442, and Asp 242, when bound to compound **7g**. The analysis of the RMSF trajectory revealed minimal fluctuations for atomic indices 1 to 35, indicating a stable interaction between compound **7g** and α-glucosidase. In contrast, the RMSF of acarbose demonstrated greater fluctuations throughout the simulation, suggesting lower stability compared to compound **7g**. Acarbose reached the highest fluctuation at 3.0 Å, further supporting the conclusion that the α-glucosidase–compound **7g** complex is more stable than the α-glucosidase–acarbose complex.

##### Molecular Dynamics Simulation of α-Amylase with Compound **7g** and Acarbose

**Figure 10:**
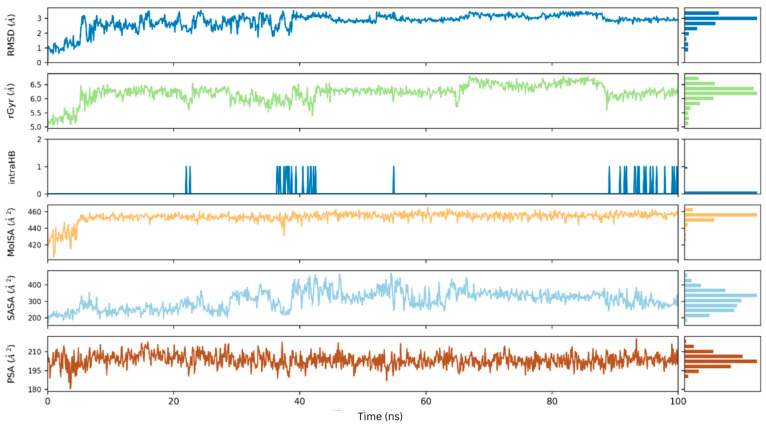
Trajectories depicting the different properties—RMSD (Å), the radius of gyration, intra hydrogen bonds, molecular surface area, solvent accessible surface area, and the polar surface area of compound **7g** when complexed with α-amylase.

The outcomes of the MD simulation in Figure 10 were in accordance with the previous study by Mohamed et al., 2023 [24].

The RMSD of the system ranges from 0 Å to 3 Å, indicating that no significant structural alterations are occurring. A prominent peak in the histogram at approximately 2 Å suggests that the structure maintains stability with minimal deviation from this value. The radius of gyration remains relatively stable between 5.5 Å and 6.5 Å; however, at 40 ns, the RMSD becomes almost constant, which is evident from the trajectory. Furthermore, a peak in the histogram at approximately 6 Å indicates that the protein consistently maintains a level of compactness. Intramolecular hydrogen bonding is generally low, averaging between 0 and 1, without any increments. This finding suggests that the hydrogen bonding network is comparatively low. The histogram illustrates that lower hydrogen bond counts are more prevalent. The molecular surface area fluctuates between 430 Å^2^ and 470 Å^2^, indicating variability in the exposure of surface residues. A histogram peak at approximately 450 Å^2^ signifies that this level of surface exposure represents the predominant state. Over time, the molecule exhibits an increasing exposure to the solvent, as evidenced by the upward trend in SASA, particularly in the latter half of the simulation. This observation is further corroborated by a histogram peak at 400 Å^2^. As the exposure of polar residues varies, the PSA ranges from 510 Å^2^ to 570 Å^2^, with a histogram peak near 540 Å^2^, suggesting that this level of polar surface exposure is most common. Collectively, these results indicate that the α-amylase–ligand complex maintains stability throughout the simulation. Though the number of hydrogen bonds was one, the degree of the formation of hydrogen bonds was high at the end of the simulation, thus maintaining the stability of the ligand with the protein. The key scientific factors contributing to the differences in the number of hydrogen bonds observed between molecular docking and molecular dynamics simulations include the contrasting static and the dynamic nature of the methods, the level of conformational sampling, the treatment of solvent effects, the time scale of observations, the accuracy of energy calculations, and the consideration of environmental and flexibility factors. Molecular dynamics provides a more comprehensive and realistic perspective on hydrogen bonding patterns, due to its dynamic nature and thorough sampling of molecular interactions over time.

**Figure 11:**
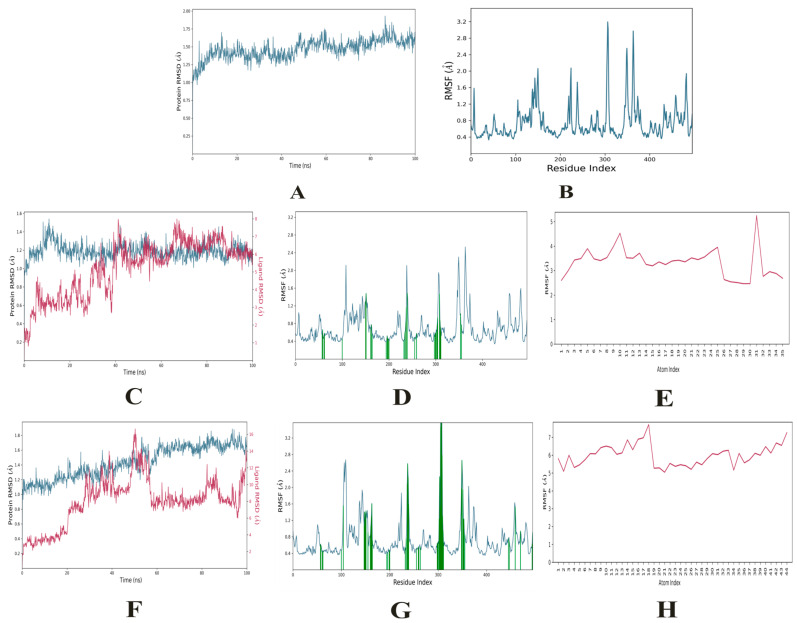
MD trajectories of α-amylase complexed with compound **7g** and acarbose. (**A**) RMSD of α-amylase. (**B**) RMSF of α-amylase. (**C**) RMSD of α-amylase–compound **7g**. (**D**) RMSF of α-amylase (**E**) RMSF of compound **7g**; (**F**) RMSD of α-amylase–acarbose (**G**) RMSF of α-amylase (**H**) RMSF of acarbose. The pink color in Figures (**C**,**F**) indicates the RMSD of compound **7g** and acarbose, and the blue color indicates the protein (α-glucosidase) RMSD. The blue color in Figures (**B**,**D**,**G**) indicates the RMSF of the protein i.e., in this case, it is α-glucosidase. The pink color in Figures (**E**,**H**) indicates the RMSF of the compound **7g** and acarbose respectively. The green color in Figures (**D**,**G**) indicates Proteins (amino acid residues) that interact with the ligand, which are marked with green-colored vertical bars.

The results from the MD simulation study align closely with the findings of Mohammed et al. (2023) [24]. Their research indicated that the RMSD of the unbound enzyme significantly increased during the first 10 ns, reaching a peak of 1.7 Å, followed by stable fluctuations over the next 30 ns (Figure 11). Ultimately, the RMSD stabilized at 1.8 Å during the final 15 ns of the simulation. In contrast, the RMSD values for both the α-amylase–acarbose complex and the α-amylase–compound **7g** complex show that compound **7g** showed consistent stability throughout the simulation period compared to acarbose. In this study, the RMSD value for the apoprotein also peaked at 1.7 Å, while the α-amylase–compound **7g** complex and the α-amylase–acarbose complex exhibited RMSD values ranging from 1.0 Å to 1.2 Å. Notably, although acarbose initially bound to the α-amylase binding site, its stability decreased after approximately 60 to 80 ns of the simulation. Regarding the root mean square fluctuation (RMSF), compound **7g** exhibited its highest fluctuation at 5 Å, whereas acarbose demonstrated even greater fluctuation, peaking at 7 Å. Compound **7g** interacted with crucial catalytic site residues of α-amylase, including Asp 197, Glu 233, and Asp 300. The analysis of the RMSF trajectory showed minimal fluctuations for atomic indices ranging from 1 to 35, indicating a stable interaction between compound **7g** and α-amylase. In contrast, the RMSF values for acarbose exhibited greater variability throughout the simulation, suggesting reduced stability compared to compound **7g**. These findings highlight that the α-amylase–compound **7g** complex exhibits superior stability relative to the α-amylase–acarbose complex.

##### Molecular Dynamics Simulation of Aldose Reductase with Compound **7g** and Quercetin

**Figure 12:**
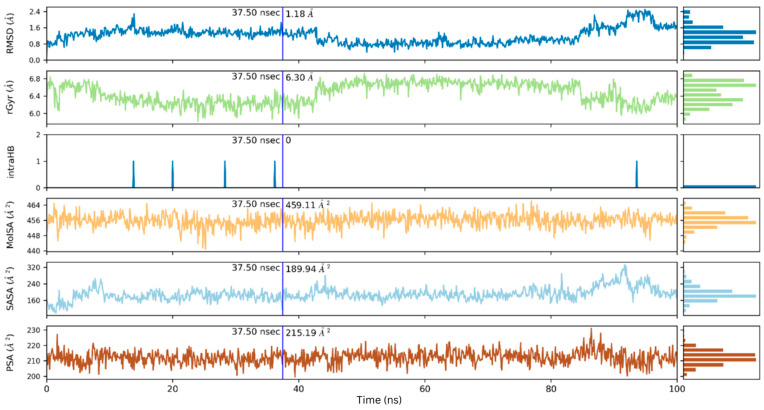
Trajectories depicting the different properties—RMSD (Å), the radius of gyration, intra hydrogen bonds, molecular surface area, solvent accessible surface area, and the polar surface area of compound **7g** when complexed with aldose reductase.

The trajectories depicting the ligand properties of compound **7g**–aldose reductase is represented in Figure 12. The RMSD ranges from 0.8 Å to 2.4 Å, indicating fluctuations in the structural stability. A notable transition is marked at 37.50 ns, with the RMSD at 1.18 Å.

**Figure 13:**
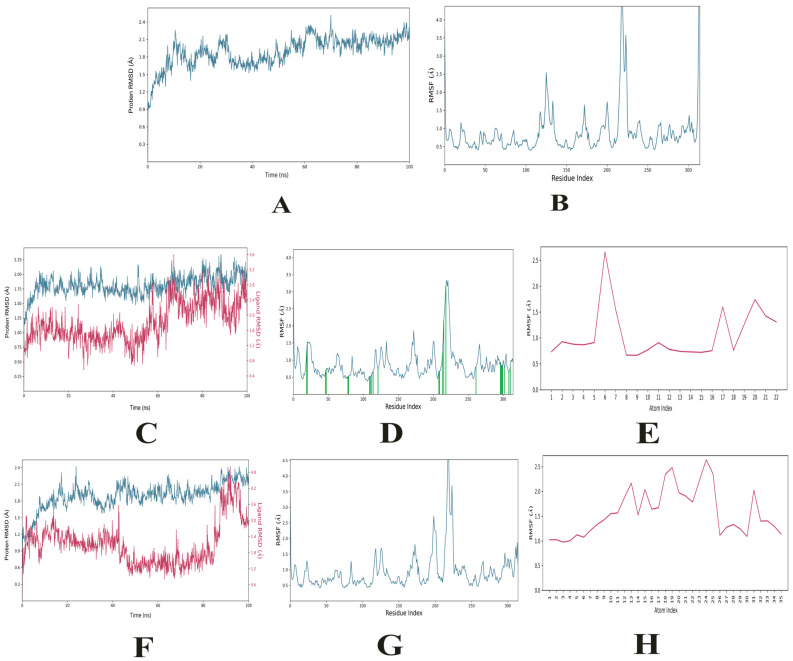
MD trajectories of aldose reductase complexed with compound **7g** and quercetin. (**A**) RMSD of aldose reductase. (**B**) RMSF of aldose reductase. (**C**) RMSD of aldose reductase–compound **7g**. (**D**) RMSF of aldose reductase. (**E**) RMSF of compound **7g**. (**F**) aldose reductase–quercetin RMSD. (**G**) RMSF of aldose reductase. (**H**) RMSF of quercetin. The pink color in Figures (**C**,**F**) indicates the RMSD of compound **7g**, and the blue color indicates the protein RMSD. The blue color in Figures (**B**,**D**,**G**) indicates the RMSF of the protein i.e., in this case, it is aldose reductase. The pink color in Figures (**E**,**H**) indicates the RMSF of the compound **7g** and quercetin respectively. The green color in Figure (**D**) indicates Proteins (amino acid residues) that interact with the ligand, which are marked with green-colored vertical bars.

The structural stability of the examined system is evident, with occasional fluctuations highlighted by the histogram, which exhibits a prominent peak at approximately 1.8 Å. The radius of gyration remains relatively constant between 6.0 Å and 6.8 Å, with a noteworthy measurement of 6.30 Å, recorded at 37.50 ns. This observation is further supported by the histogram, which demonstrates a narrow distribution around 6.3 Å, indicative of consistent compactness throughout the duration of the simulation. Intramolecular hydrogen bonds are notably low, typically varying from zero to one. At the specific time point of 37.50 ns, the observation of zero hydrogen bonds was made. The histogram suggests a restricted hydrogen bonding network, predominantly reflecting states characterized by a lack of hydrogen bonds or very few present. The molecular surface area is quantified at 459.11 Å^2^ and exhibits fluctuations between 448 Å^2^ and 466 Å^2^. The histogram peaks at 459 Å^2^, indicating a prevalent state of surface exposure. An identifiable increase in solvent exposure was observed over time, as reflected in the overall upward trend of the SASA, particularly towards the simulation’s conclusion, where a value of 189.94 Å^2^ was recorded. Additionally, the peak in the histogram at 200 Å^2^ corroborates the dynamics of escalating solvent exposure. The PSA ranges from 210 Å^2^ to 230 Å^2^, correlating with variations in the exposure of polar residues, and the PSA was measured at 215.19 Å^2^. The histogram peak at approximately 215 Å^2^ indicates that the prevailing state is one of enhanced polar surface exposure. These findings are consistent with earlier research conducted by Antony and Vijayan (2015) [25]. Overall, the data support the conclusion that the ligand–aldose reductase complex exhibits significant stability. Figure 13 represents the MD simulation analysis trajectories.

The outcomes of the molecular dynamics simulation corroborated the findings reported by Antony and Vijayan (2015) [25]. In their study, the RMSD of the unbound enzyme exhibited rapid growth characterized by oscillations until reaching 10 ns, peaking at 1.5 Å, followed by stable oscillations for the subsequent 30 ns. The enzyme achieved stabilization at an RMSD of 2.0 Å during the final 15 ns of the simulation. Conversely, the complexes formed between aldose reductase and compound **7g**, as well as the aldose reductase–quercetin complexes, demonstrated decreased RMSD values, indicating that these bound-state enzymes-maintained stability throughout the simulation. These results suggest that the target–ligand complexes attained equilibrium during the simulation period. The RMSD value of the apoprotein aldose reductase was 2.1 Å. In comparison, the RMSD of the aldose reductase–compound **7g** complex demonstrated an RMSD of 1.0 Å (for aldose reductase) and 2.4 Å (for the compound **7g**–aldose reductase complex), and concerning the aldose reductase–acarbose complex, apoprotein had an RMSD of 2.1 Å and 3.0 Å (for α-glucosidase–acarbose). In terms of the RMSF, both quercetin and compound **7g** displayed the highest fluctuation, at 2.5 Å. Furthermore, the residues Tyr 309, Cys 298, Trp 20, Val 47, and Trp 111, as well as compound **7g**, interacted with aldose reductase. The RMSF trajectory indicated minimal variation in the atomic indices from 1 to 35, signifying that compound **7g** exhibited stability in its association with aldose reductase. In contrast, the aldose reductase–acarbose complex displayed more pronounced oscillations (from atomic indices 1 to 22) from the outset of the simulation, suggesting reduced stability compared to compound **7g**. These findings indicate that the aldose reductase–compound **7g** complex is generally more stable than the aldose reductase–quercetin combination.

### 2.4. Physicochemical and Absorption, Distribution, Metabolism, Excretion, and Toxicity (ADMET) Properties

**Figure 14:**
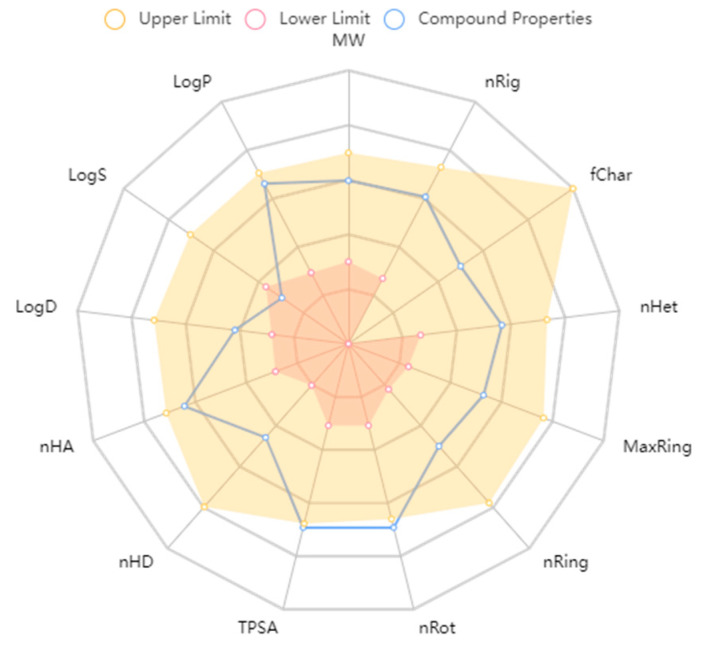
ADMET radar analysis of compound **7d**.

Table 3 indicates that compound **7g** complies with both Lipinski’s Rule of Five and Pfizer’s rule. These characteristics suggest that **7g** is an ideal candidate for oral administration (Figure 14). Additionally, its structural characteristics, such as the number of H-bond donors and acceptors, rotatable bonds, molecular flexibility, and topological polar surface area, further support its potential as an orally administered drug.

For ADMET properties, compound **7g** showed favorable absorption values in the Caco-2 and MDCK cell lines, and no Pgp inhibitor was measured. Its distribution volume score suggests suitable distribution within the human body. Importantly, **7g** does not pass the blood–brain barrier. Its clearance value of 13 mL/kg/min indicates efficient elimination from the body post-pharmacological action. Furthermore, assessments for AMES toxicity and acute toxicity parameters predict that **7g** is non-toxic to humans. These findings suggest that **7g** is a distinguished candidate for further clinical trial studies. This aligns with our previous research, where compounds such as Isoindoline and rutin were also deemed suitable for oral consumption [16].

### 2.5. Structure–Activity Relationship

**Figure 15:**
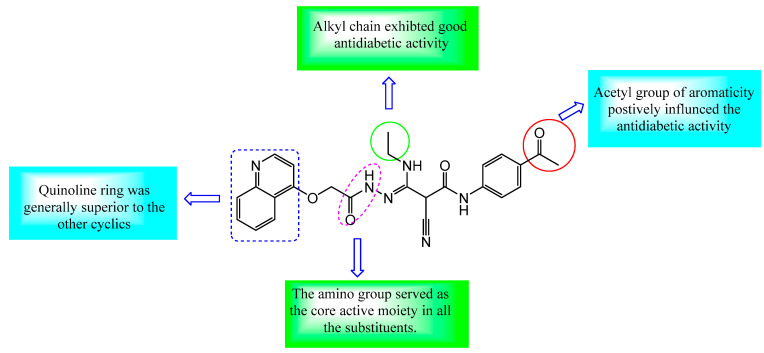
The initial examination of the SAR of the derivatives **5a**–**g**.

In the structure–activity relationship (SAR), for the inhibition of carbohydrate-digesting enzymes (α-glucosidase, α-amylase, and aldose reductase), all the substances exhibit significant antidiabetic effects in comparison to the standard drugs, acarbose and quercetin. The addition of bicyclic rings, such as quinoline and naphthalene, enhances the **5a**,**b** activity of the parent molecules. The quinoline derivatives exhibited the superior inhibition of all the examined enzymes compared to other derivatives, with the exception of compounds **7d** and **7f** (containing a naphthalene ring), which had more activity in aldose reductase than compounds **7b** and **7e** (containing a quinoline ring). From Table 1, we observed that compound **7g** had enhanced inhibitory effects relative to the other compounds (**7a**–**g**). The structure of compound **7g** has improved beyond the parent’s compounds through the coupling of a quinoline ring to the primary structure, the attachment of a new alkyl group to the amine core, and the substitution of the amide group on the benzene ring with an acyl group (Figure 15). The modification of compound **5g** may significantly enhance enzyme binding, as evidenced by lower IC_50_ values across all the assays. Compound **7g** outperformed the conventional inhibitors acarbose and quercetin, indicating that the structural modifications boost inhibition by facilitating stronger contacts with the enzymes’ active regions.

## 3. Materials and Methods

### 3.1. Reagents and Instrumentation

An Infrared (IR) analysis (ν_max_, cm^−1^) was performed by Fourier Transform–Infrared (FT-IR) Perkin Elmer, using KBr. Furthermore, ^1^H, 13CNMR spectra (400, 100 MHz) were achieved using Avance AV 3HD in DMSO-d_6_. Elemental analyses were detected on the LECO Truspec Micro Analyzer.

### 3.2. Synthesis

#### 3.2.1. Synthesis of Compounds **3a**,**b**

*p*-Amino/methylacetophenone **1a**,**b** (5 g, 37 mmol) in 35 mL of DMF was refluxed with ethyl 2-cyanoacetate (4.18 g, 37 mmol) for 16 hr. The product was filtered and subsequently crystallized from ethanol [26].

#### 3.2.2. Synthesis of Compounds **5a**–**d**

To a solution of **3a**,**b** (37 mmol) in DMF (20 mL), a cold suspension of finely powdered KOH (2 g, 35 mmol) in dimethyl formaldehyde (DMF, 5 mL) and phenyl/ethyl isothiocyanate (37 mmol) were added and stirred for 12 hr at RT. Then, dimethyl sulfate (4.66 mL, 37 mmol) was added and stirred for an extra 5 hr and was poured over ice. The solid product that precipitated was filtered, dried, and subjected to recrystallization from a mixture of EtOH:DMF (2:1) [27].

#### 3.2.3. Synthesis of Compounds **7a**–**h**

Into 20 mL of (2 mmol) **5a**–**d** solution in ethanol, an equimolar amount of substituted naphthalene/quinoline-acetohydrazides **6a**,**b** (2 mmol) and 1 mL piperidine were added, and the mixture was refluxed for 8 hr. The formed solid in the ice layer was filtered and recrystallized from ethanol to yield compounds **7a**–**g**.

4-(2-cyano-3-(2-(2-(naphthalen-2-yloxy)acetyl)hydrazineylidene)-3-(phenylamino) propanamido)benzamide **7a**

Yield, 81%; yellow solid; mp: 127–129 °C; **IR**: 1554 (C=C aromatic), 1608 (C=N), 1682 (br, 2C=O), 2191 (CN), 2995 (CH aliphatic), 3032 (CH aromatic), 3419, 3345, 3314, 3134 (NH_2_, 3NH); **^1^H NMR**: δ 11.59 (s, 1H, NH), 9.77 (s, 2H, NH), 7.86–7.83(m, 4H, Ar), 7.81–7.79 (m, 3H, Ar), 7.7.76–7.74 (m, 6H, Ar), 7.60 (d, *J* = 14.8, 2H), 7.39–7.37 (m, 1H, Ar), 7.29–7.25 (m, 1H, Ar),7.21 (s, 2H, NH_2_), 4.62 (s, 2H, CH2). **^13^C NMR** δ: 167.83, 134.49, 129.71, 128.48, 128.00, 127.16, 126.96, 124.30, 124.06, 120.28, 119.14, 119.03, 107.51, 66.78, 66.60; **MS**: 522 (M^+^); Anal. calcd. for C_29_H_24_N_6_O_4_: C, 66.88; H, 4.79; N, 16.10. Found: C, 66.73; H, 4.53; N, 16.13.

4-(2-cyano-3-(phenylamino)-3-(2-(2-(quinolin-4-yloxy)acetyl)hydrazineylidene) propanamido)benzamide **7b**

Yield, 84%; yellow solid; mp: 112–114 °C; IR: 1556 (C=C aromatic), 1626 (C=N), 1679 (br, 2C=O), 2191 (CN), 2995 (CH aliphatic), 3032 (CH aromatic), 3418, 3353, 3332, 3135 (NH_2_, 3NH); **^1^H NMR**: δ 11.59 (s, 1H, NH), 9.77 (s, 1H, NH), 7.84–7.20 (m, 15H, Ar), 7.38(s, 2H, NH_2_), 4.75 (s, 2H, CH_2_), 4.30 (s, 1H, CH). **^13^C NMR**: δ 167.84, 143.11, 129.84, 129.70, 128.50, 124.07, 123.02, 121.15, 120.26,118.83, 78.84. **MS**: 522 (M^+^); Anal. calcd. for C_28_H_23_N_7_O_4_: C, 64.48; H, 4.45; N, 18.80. Found: C, 64.22; H, 4.36; N, 18.59.

4-(2-cyano-3-(ethylamino)-3-(2-(2-(naphthalen-2-yloxy)acetyl)hydrazineylidene) propanamido)benzamide **7c**

Yield, 69%; brown solid; mp: 131–133 °C; IR: 1513 (C=C aromatic), 1626 (C=N), 1679 (br, 2C=O), 2197 (CN), 2995 (CH aliphatic), 3032 (CH aromatic), 3489, 3301, 3265, 3185 (NH_2_, 3NH); **^1^H NMR**: δ 10.12 (s, 1H, NH), 9.41(s, 1H, NH), 9.03 (s, 1H, NH), 7.82–7.23 (m, 15H, Ar), 7.38 (s, 2H, NH_2_), 4.77 (s, 2H, CH_2_), 4.76 (s, 1H, CH), 2.65 (s, 2H, CH_2_), 1.21(s, 3H, CH_3_). **^13^C NMR**: δ 170.68, 167.95, 167.88, 156.06, 142.87, 141.86, 134.53, 129.82, 129.27, 128.83, 128.48, 128.01, 127.25, 126.96, 124.37, 120.19, 119.84, 119.14, 107.82, 107.72, 75.18, 66.63, 18.11, 15.82. **MS**: 487 (M^+^); Anal. calcd. for C_26_H_26_N_6_O_4_: C, 64.19; H, 5.39; N, 17.27. Found: C, 64.03; H, 5.18; N, 17.21.

*N*-(4-acetylphenyl)-2-cyano-3-(2-(2-(naphthalen-2-yloxy)acetyl)hydrazineylidene)-3-(phenylamino)propenamide **7d**

Yield, 72%; brown solid; mp: 143–145 °C; IR: 1560 (C=C aromatic), 1625 (C=N), 1653 (br, 2C=O), 2202 (CN), 3275, 3205, 3170 (3NH); **^1^H NMR**: δ 11.58 (s, 1H, NH), 10.09 (s, 1H, NH), 9.71(s, 1H, NH), 8.27–7.22 (m, 16H, Ar), 4.75 (s, 2H, CH_2_), 2.26 (s, 3H, CH_3_). **^13^C NMR:** δ 167.62, 164.64, 139.08, 138.17, 131.69, 131.69, 129.70, 129.70, 124.01, 123.36, 123.36, 79.63, 16.88. **MS**: 520 (M^+^); Anal. calcd. for C_30_H_25_N_5_O_4_: C, 69.35; H, 4.85; N, 13.48. Found: C, 69.25; H, 4.68; N, 13.30.

*N*-(4-acetylphenyl)-2-cyano-3-(phenylamino)-3-(2-(2-(quinolin-4-yloxy)acetyl) hydrazineylidene)propenamide **7e**

Yield, 81%; yellow solid; mp: 106–108 °C; **IR**: 1558 (C=C aromatic), 1626 (C=N), 1665 (br, 2C=O), 2196 (CN), 2995 (CH aliphatic), 3032 (CH aromatic), 3384, 3225, 3190 (3NH); **^1^H NMR** δ 11.58 (s, 1H, NH), 10.17 (s, 1H, NH), 9.71(s, 1H, NH), 8.91–7.21 (m, 15H, Ar), 4.89 (s, 2H, CH_2_), 2.26 (s, 3H, CH3). **^13^C NMR**: δ 167.63, 164.63, 154.39, 149.79, 139.11, 138.17, 136.49, 131.85, 131.70, 129.70, 127.21, 126.48, 124.02, 123.36, 122.47, 121.49, 115.97, 68.34, 16.89. **MS**: 522 (M^+^); Anal. calcd. for C_29_H_24_N_6_O_4_: C, 66.87; H, 4.59; N, 16.21. Found: C, 66.72; H, 4.51; N, 16.03.

*N*-(4-acetylphenyl)-2-cyano-3-(ethylamino)-3-(2-(2-(naphthalen-2-yloxy)acetyl) hydrazineylidene)propenamide **7f**

Yield, 72%; bright yellow; mp: 115–117 °C; **IR**: 1567 (C=C aromatic), 1626 (C=N), 1678,1656 (br, 2C=O), 2194 (CN), 2981 (CH aliphatic), 3028 (CH aromatic), 3312, 3214, 3155 (3NH); **^1^H NMR**: δ 10.54 (s, 1H, NH), 9.42 (s, 1H, NH), 9.35 (s, 1H, NH), 7.86–7.25 (m, 11H, Ar), 4.61(s, 2H, CH_2_), 3.56 (d, *J* = 1.2 Hz, 2H), 3.55 (s, CH, 1H), 2.64 (s, 3H, CH_3_), 1.18 (s, 3H, CH_3_). **^13^C NMR**: δ 170.55, 166.98, 166.24, 156.16, 138.48, 134.50, 131.64, 129.76, 129.19, 128.00, 127.20, 126.92, 124.30, 123.36, 119.81, 119.22, 115.62, 107.51, 75.09, 66.79, 18.09, 15.80. **MS**: 472 (M^+^); Anal. calcd. for C_26_H_25_N_5_O_4_: C, 66.34; H, 5.44; N, 14.95. Found: C, 66.05; H, 5.32; N, 14.66.

*N*-(4-acetylphenyl)-2-cyano-3-(ethylamino)-3-(2-(2-(quinolin-4-yloxy)acetyl) hydrazineylidene)propenamide **7g**

Yield, 78%; yellow solid; mp: 119–121 °C; **IR**: 1570 (C=C aromatic), 1620 (C=N), 1664,1652 (br, 2C=O), 2194 (CN), 2968 (CH aliphatic), 3053 (CH aromatic), 3303, 3222, 3157 (3NH); **^1^H NMR**: δ 10.54 (s, 1H, NH), 9.35 (s, 1H, NH), 7.55–7.43 (m, 10H, Ar), 4.86 (s, 2H, CH_2_), 4.84 (s, 2H, CH_2_), 3.56 (dd, *J* = 7.1, 5.8 Hz, 2H), 3.54 (s, 1H, CH), 2.64 (s, 3H, CH_3_), 1.18 (t, *J* = 7.2 Hz, 3H). **^13^C NMR**: δ 170.55, 166.24, 138.48, 134.51, 131.85, 131.64, 127.84, 127.04, 125.78, 123.36, 119.81, 115.61, 75.09, 18.09, 15.81. **MS**: 472 (M^+^); Anal. calcd. for C_26_H_25_N_5_O_4_: C, 66.24; H, 5.29; N, 14.90. Found: C, 66.13; H, 5.36; N, 14.67.

### 3.3. Biology

#### In Vitro Carbohydrate Enzyme Inhibition Assays

Using different doses (0–1000 μg/mL) dissolved in dimethyl sulphoxide (DMSO), the synthetic compounds **7a**–**g** were examined for their capacity to inhibit diabetes-related enzymes. Quercetin and acarbose were used as positive controls for particular enzymes. Porcine pancreatic amylase, yeast α-glucosidase, and human recombinant aldose reductase were tested for enzyme inhibition using various substrates, such as starch (1%), pNPG (0.5 mM), and DL-glyceraldehyde (5 mM) for each enzyme [23]. By comparing changes in the test sample absorbance levels to control values and computing inhibition percentages using the following formula, the degree of inhibition was ascertained:

Inhibition  (%) = [A×(control)] − [{A × (sample)}/{A × (control)} × 100].

The inhibitory effects of the compounds were assessed using a least-squares regression analysis, correlating logarithmic concentrations with the percentage of inhibition, providing IC_50_ values (concentrations that inhibit enzyme activity by 50%). These IC_50_ values are the concentrations of test substances needed to provide 50% inhibition, compared to controls [16].

### 3.4. Computational Studies

#### 3.4.1. Molecular Docking Studies

The Biologics suite of Schrodinger was used in the structure-based molecular docking study to evaluate the antidiabetic activity of the synthesized derivatives (**7a**–**g**) against three target proteins. The X-ray structures of α-glucosidase for (PDB ID: 3A4A), α-amylase for (PDB-ID 1DHK), and aldose reductase for (PDB ID: 1IEI) were used in the molecular docking investigation. The structures were retrieved from the Research Collaboratory for Structural Bioinformatics’ Protein Data Bank (RCSB-PDB), which is available online at (www.rcsb.org, accessed on 10 April 2002). The proteins were prepared using the Maestro 13.9 platform (Schrödinger, Inc. 2024). The ligands and the standards were drawn using a 2D sketcher, followed by ligand preparation using the Ligprep module of the Maestro. The energy was then processed using the OPLS force field (LigPrep, Schrödinger, Inc., New York, NY, USA, 2024). In molecular docking simulations and computational chemistry, “processing energy using the OPLS force field” involves calculating and minimizing the potential energy of a molecular system, based on the OPLS (Optimized Potentials for Liquid Simulations) parameters. These parameters describe bond lengths, angles, dihedrals, Van der Waals interactions, and electrostatic interactions. The process starts with calculating bond stretching energy, followed by angle bending, torsional, Van der Waals, and electrostatic energy. After determining these components, the total potential energy is minimized to alleviate steric clashes or unfavorable interactions in the initial configuration. Once minimized, the system undergoes equilibration steps, in which temperature and pressure are gradually adjusted using molecular dynamics (MDs) simulations, ensuring stability before collecting the data for analysis. The protein targets were prepared using the Protein Preparation Wizard. After protein preparation, in a grid generation relative to α-glucosidase, the inhibitor binding site of the co-crystallized ligand GLC, relative to α-amylase, the key amino acids Glu 233, Asp 197, and Asp 300, which are found in the literature, were specified in the receptor grid generation, and finally, concerning aldose reductase, the co-ordinates of the co-crystal ligand were used for the docking of the ligands. Using Maestro 13.9’s Glide XP docking module, the prepared ligands were docked inside a grid box of 30 Å (Glide, Schrödinger, Inc. 2024, New York, USA). The docking precision was configured in a flexible mode for the docking procedure. To analyze and validate the results, we compared the computations with those of the standard molecules after the docking results were displayed using Maestro.

The molecular docking was validated by removing the co-crystal ligands of α-glucosidase (GLC 601) and aldose reductase (Zenerastat). Subsequently, the respective co-crystal ligands were re-docked with their respective targets using the above-mentioned procedure.

#### 3.4.2. Molecular Dynamics Simulations

The MD simulations were carried out by the Schrödinger LLC Desmond simulation package [28]. For all runs, the NPT ensemble was used at 300 K and 1 bar. For ligands **7a**–**g**, the relaxation time was 1 ps, and the simulation lasted 100 ns. All the simulations were conducted using the OPLS_2005 force field settings (Schrodinger Release 2024-1: Desmond Molecular Dynamics System). The particle mesh Ewald approach (Toukmaji et al., 1996) was utilized to compute the long-range electrostatic interactions [29]. In Coulomb interactions, 9.0 Å was the cutoff radius. The simplified point charge model (Zielkiewicz, J., 2005) was used to clearly define the water molecules [30,31]. With the help of an r-RESPA integrator, the nonbonded forces were computed, and every three steps it was updating the long-range forces and the short-range forces. At 4.8 ps intervals, the trajectories were stored for analysis. The Desmond MD package’s Simulation Interaction Diagram tool was used to investigate the behavior and interactions between the ligands and proteins. The stability of the MD was evaluated by analyzing the root RMSD of both the ligand and protein atom positions throughout the simulation period. Over time, the stability of the MD simulations was assessed by analyzing the RMSDs of the ligand and protein atom locations.

## 4. Conclusions

In conclusion, we effectively synthesized numerous bicyclic derivatives, which include quinoline and naphthalene rings, by diverse synthetic approaches, including the condensation reactions of quinoline and naphthalene hydrazides with chalcone derivatives. Among the compounds, **7g** distinguished itself as a premier lead candidate, demonstrating the enhanced inhibition of α-glycosidase (IC_50_; 20.23 ± 1.10 µg/mL, 42.90 µM), α-amylase (IC_50_; 17.15 ± 0.10 µg/mL), and aldose reductase (IC_50_; 12.15 ± 0.24 µg/mL, 25.77 µM), relative to common medications such as acarbose and quercetin, representing its potential as an antidiabetic agent. Compound **7g** exhibited robust enzyme binding and stability, as validated by the molecular docking and dynamics simulations, and showed no hazardous effects in the ADMET study. These data indicate its potential for further advancement as an oral antidiabetic agent.

## Figures and Tables

**Table 1:** In vitro α-glucosidase, α-amylase, and aldose reductase inhibition assays.

Compounds	Structures	IC_50_ (µg/mL)		
		Enzymes		
		α-Glucosidase	α-Amylase	Aldose Reductase
**5a**	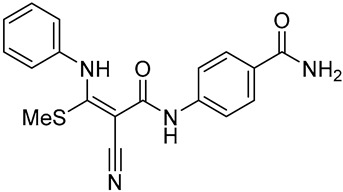	27.00 ± 0.80	33.13 ± 0.19	25.14 ± 0.30
**7a**	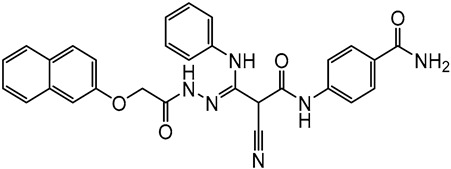	26.50 ± 0.98	22.42 ± 0.37	22.17 ± 1.10
**7b**	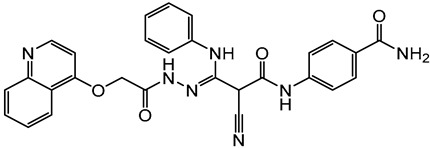	26.05 ± 1.12	19.45 ± 0.27	21.15 ± 1.00
**7c**	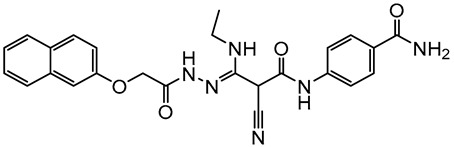	29.45 ± 2.10	26.99 ± 0.40	24.02 ± 0.32
**7d**	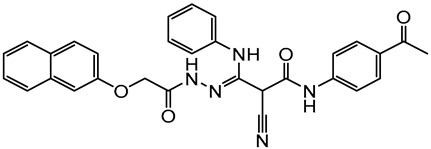	33.00 ± 0.25	23.75 ± 0.55	17.45 ± 0.10
**7e**	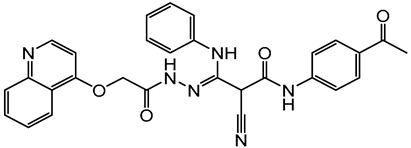	26.50 ± 0.39	19.98 ± 1.15	22.39 ± 0.37
**7f**	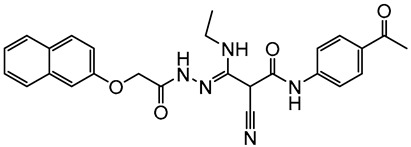	26.66 ± 0.11	29.12 ± 0.88	20.18 ± 0.84
**7g**	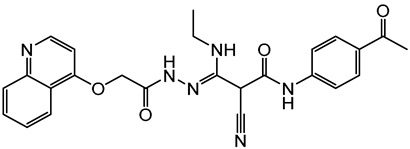	20.23 ± 1.10	17.15 ± 0.30	12.15 ± 0.24
**Acarbose**	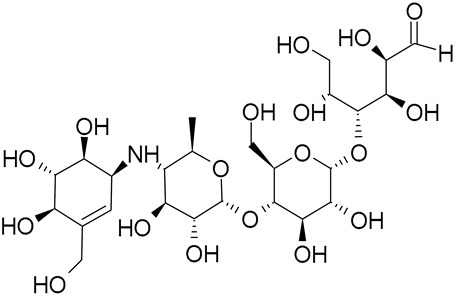	28.12 ± 0.20	25.42 ± 0.10	-
**Quercetin**	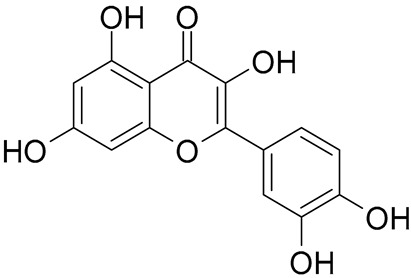	-	-	15.45 ± 0.32

Values are expressed as mean ± SE.

**Table 2:** Outcomes of molecular docking of derivatives **7a**–**g**.

Enzymes		α-Glucosidase		α-Amylase		Aldose Reductase	
	Compound	Glide Score (kcal/mol)	Number of Hydrogen Bonds	Glide Score (kcal/mol)	Number of Hydrogen Bonds	Glide Score (kcal/mol)	Number of Hydrogen Bonds
**5a**		−7.03	3	−6.28	2	−8.96	3
**7a**		−6.63	3	−6.48	2	−6.15	1
**7b**		−7.72	2	−8.23	3	−7.18	2
**7c**		−6.21	1	−7.89	2	−6.96	3
**7d**		−7.52	3	−7.02	4	−5.83	1
**7e**		−8.22	2	−6.79	3	−5.52	2
**7f**		−6.98	2	−7.55	2	−4.68	3
**7g**		−8.80	3	−8.91	3	−9.20	2
**Acarbose**		−10.87	6	−10.38	4	-	-
**Quercetin**		-	-	-	-	−9.95	3

**Table 3:** Physicochemical characteristics of compound **7g**.

Physicochemical Parameters	Values	ADMET Parameters	Values
Molecular weight (M.Wt) (g/mol)	472.19	Absorption	
Volume (L/kg of body weight)	479.518	Caco-2 permeability	−5.059 cm/s
Density (g/L)	0.985	MDCK permeability	6 × 10^−6^ cm/s
Number of hydrogen bond acceptors (nHA)	10	Pgp inhib	0.604
Number of hydrogen bond donors (nHD)	3	Distribution	
nRot	9	VD	0.693
nRing	3	Blood–brain barrier (BBB)	0.626 cm/s
nHet	10	Metabolism	
fChar	0	CYP1A2	-
nRig	22	CYP2C19	-
Flexibility	0.545	CYP2C9	-
Topological polar surface area (TPSA) (Å^2^)	125.57	CYP2D6	-
logS	−4.953 mol/L	Excretion	
logP	2.678 mol/L	Clearance	13 mL/kg/min
logD	1.627 mol/L	Toxicity	
Stereo centers	1	hERG blockers	0.007 µM
Lipinski’s rule	Accepted	AMES toxicity	0.895
Pfizer’s rule	Accepted	Carcinogenicity	0.33
Pan assay interference compounds (PAINS)	0 alerts	Acute toxicity rule	0.65
Aggregates	0 alerts		

## Data Availability

The original contributions presented in the study are included in the article/Supplementary Material, further inquiries can be directed to the corresponding authors.

## Supplementary Materials

The following supporting information can be downloaded at https://www.mdpi.com/article/10.3390/ph17121692/s1: IR, ^1^HNMR, and ^13^CNMR for the target compounds; Table S1. In vitro α-glucosidase, α-amylase, and aldose reductase inhibition assays.
