# Supplementary material for: Novel Quinoline- and Naphthalene-Incorporated Hydrazineylidene–Propenamide Analogues as Antidiabetic Agents: Design, Synthesis, and Computational Studies
Source: Pharmaceuticals (Basel). 2024 Dec 15;17(12):1692. doi: 10.3390/ph17121692 (PMC11679724; doi:10.3390/ph17121692)

Supplementary Materials

Novel Quinoline- and Naphthalene-Incorporated Hydrazineylidene–Propenamide Analogues as Antidiabetic Agents: Design, Synthesis, and Computational Studies

Osama Alharbi [1], Wael H. Alsaedi [1], Mosa Alsehli [1], Saif. H. Althagafi [2], Hussam Y. Alharbi[3], Yazeed M. Asiri [4], Ramith Ramu [5], Mohammed Al-Ghorbani[1,6,*]

Contents: **Page**

Table S1. *In vitro* α-glucosidase, α-amylase, and aldose reductase inhibition assays **2**

IR for 4-(2-cyano-3-(2-(2-(naphthalen-2-yloxy)acetyl)hydrazineylidene)-3-(phenylamino) propanamido)benzamide **7a** **3**

^{1}H NMR for 4-(2-cyano-3-(2-(2-(naphthalen-2-yloxy)acetyl)hydrazineylidene)-3-(phenylamino) propanamido)benzamide **7a** **4**

^{13}C NMR for 4-(2-cyano-3-(2-(2-(naphthalen-2-yloxy)acetyl)hydrazineylidene)-3-(phenylamino) propanamido)benzamide **7a** **5**

IR for 4-(2-cyano-3-(phenylamino)-3-(2-(2-(quinolin-4-yloxy)acetyl)hydrazineylidene) propanamido)benzamide **7b** **6**

^{1}H NMR for 4-(2-cyano-3-(phenylamino)-3-(2-(2-(quinolin-4-yloxy)acetyl)hydrazineylidene) propanamido)benzamide **7b** **7**

^{13}C NMR for 4-(2-cyano-3-(phenylamino)-3-(2-(2-(quinolin-4-yloxy)acetyl)hydrazineylidene) propanamido)benzamide **7b** **8**

IR for 4-(2-cyano-3-(ethylamino)-3-(2-(2-(naphthalen-2-yloxy)acetyl)hydrazineylidene) propanamido)benzamide **7c** **9**

^{1}H NMR for 4-(2-cyano-3-(ethylamino)-3-(2-(2-(naphthalen-2-yloxy)acetyl)hydrazineylidene) propanamido)benzamide **7c** **10**

^{13}C NMR for 4-(2-cyano-3-(ethylamino)-3-(2-(2-(naphthalen-2-yloxy)acetyl)hydrazineylidene) propanamido)benzamide **7c** **11**

IR for *N*-(4-acetylphenyl)-2-cyano-3-(2-(2-(naphthalen-2-yloxy)acetyl)hydrazineylidene)-3-(phenylamino)propenamide **7d** **12**

^{1}H NMR for *N*-(4-acetylphenyl)-2-cyano-3-(2-(2-(naphthalen-2-yloxy)acetyl)hydrazineylidene)-3-(phenylamino)propenamide **7d** **13**

^{13}C NMR for *N*-(4-acetylphenyl)-2-cyano-3-(2-(2-(naphthalen-2-yloxy)acetyl)hydrazineylidene)-3-(phenylamino)propenamide **7d** **14**

IR for *N*-(4-acetylphenyl)-2-cyano-3-(phenylamino)-3-(2-(2-(quinolin-4-yloxy)acetyl) hydrazineylidene)propenamide **7e** **15**

^{1}H NMR for *N*-(4-acetylphenyl)-2-cyano-3-(phenylamino)-3-(2-(2-(quinolin-4-yloxy)acetyl) hydrazineylidene)propenamide **7e** **16**

^{13}C NMR for *N*-(4-acetylphenyl)-2-cyano-3-(phenylamino)-3-(2-(2-(quinolin-4-yloxy)acetyl) hydrazineylidene)propenamide **7e** **17**

IR for *N*-(4-acetylphenyl)-2-cyano-3-(ethylamino)-3-(2-(2-(naphthalen-2-yloxy)acetyl) hydrazineylidene)propenamide **7f** **18**

^{1}H NMR for *N*-(4-acetylphenyl)-2-cyano-3-(ethylamino)-3-(2-(2-(naphthalen-2-yloxy)acetyl) hydrazineylidene)propenamide **7f** **19**

^{13}C NMR for *N*-(4-acetylphenyl)-2-cyano-3-(ethylamino)-3-(2-(2-(naphthalen-2-yloxy)acetyl) hydrazineylidene)propenamide **7f** **20**

IR for *N*-(4-acetylphenyl)-2-cyano-3-(ethylamino)-3-(2-(2-(quinolin-4-yloxy)acetyl) hydrazineylidene)propenamide **7g** **21**

^{1}H NMR for *N*-(4-acetylphenyl)-2-cyano-3-(ethylamino)-3-(2-(2-(quinolin-4-yloxy)acetyl) hydrazineylidene)propenamide **7g** **22**

^{13}C NMR for *N*-(4-acetylphenyl)-2-cyano-3-(ethylamino)-3-(2-(2-(quinolin-4-yloxy)acetyl) hydrazineylidene)propenamide **7g** **23**

Table S1. In vitro α-glucosidase, α-amylase, and aldose reductase inhibition assays

**Compounds**	IC_{50} (μM)		
	**Enzymes**		
	α-Glucosidase	α-Amylase	Aldose reductase
**5a**	76.62	94.01	71.34
**7a**	52.96	44.81	44.31
**7b**	49.95	37.29	40.55
**7c**	60.53	55.48	49.37
**7d**	63.52	45.71	33.59
**7e**	50.91	38.38	43.01
**7f**	56.54	61.76	42.80
**7g**	42.90	36.37	25.77
**Acarbose**	43.59	39.41	-
**Quercetin**	-	-	51.12

IR for 4-(2-cyano-3-(2-(2-(naphthalen-2-yloxy)acetyl)hydrazineylidene)-3-(phenylamino) propanamido)benzamide **7a**

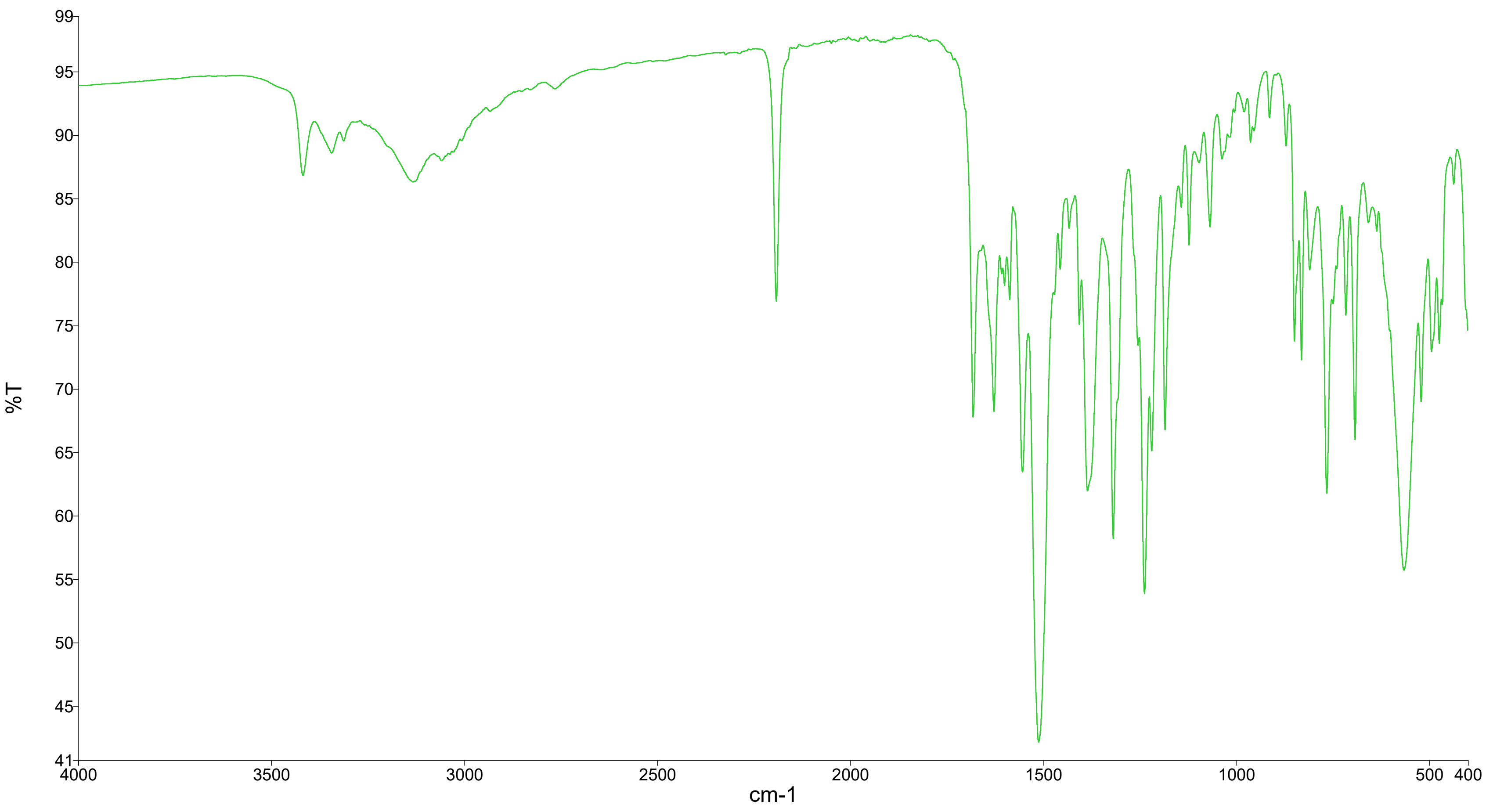

^{1}H NMR for 4-(2-cyano-3-(2-(2-(naphthalen-2-yloxy)acetyl)hydrazineylidene)-3-(phenylamino) propanamido)benzamide **7a**

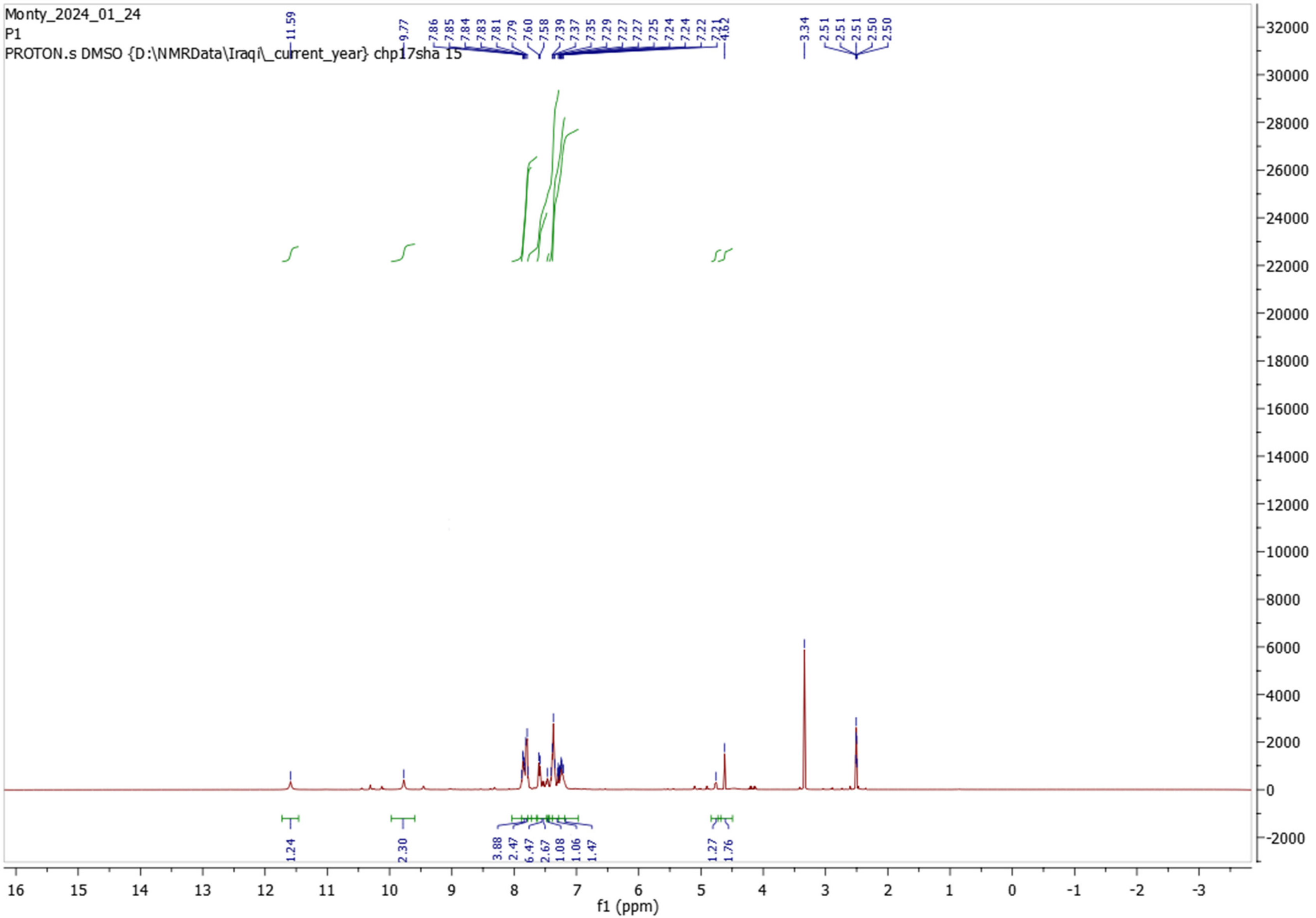

^{13}C NMR for 4-(2-cyano-3-(2-(2-(naphthalen-2-yloxy)acetyl)hydrazineylidene)-3-(phenylamino) propanamido)benzamide **7a**

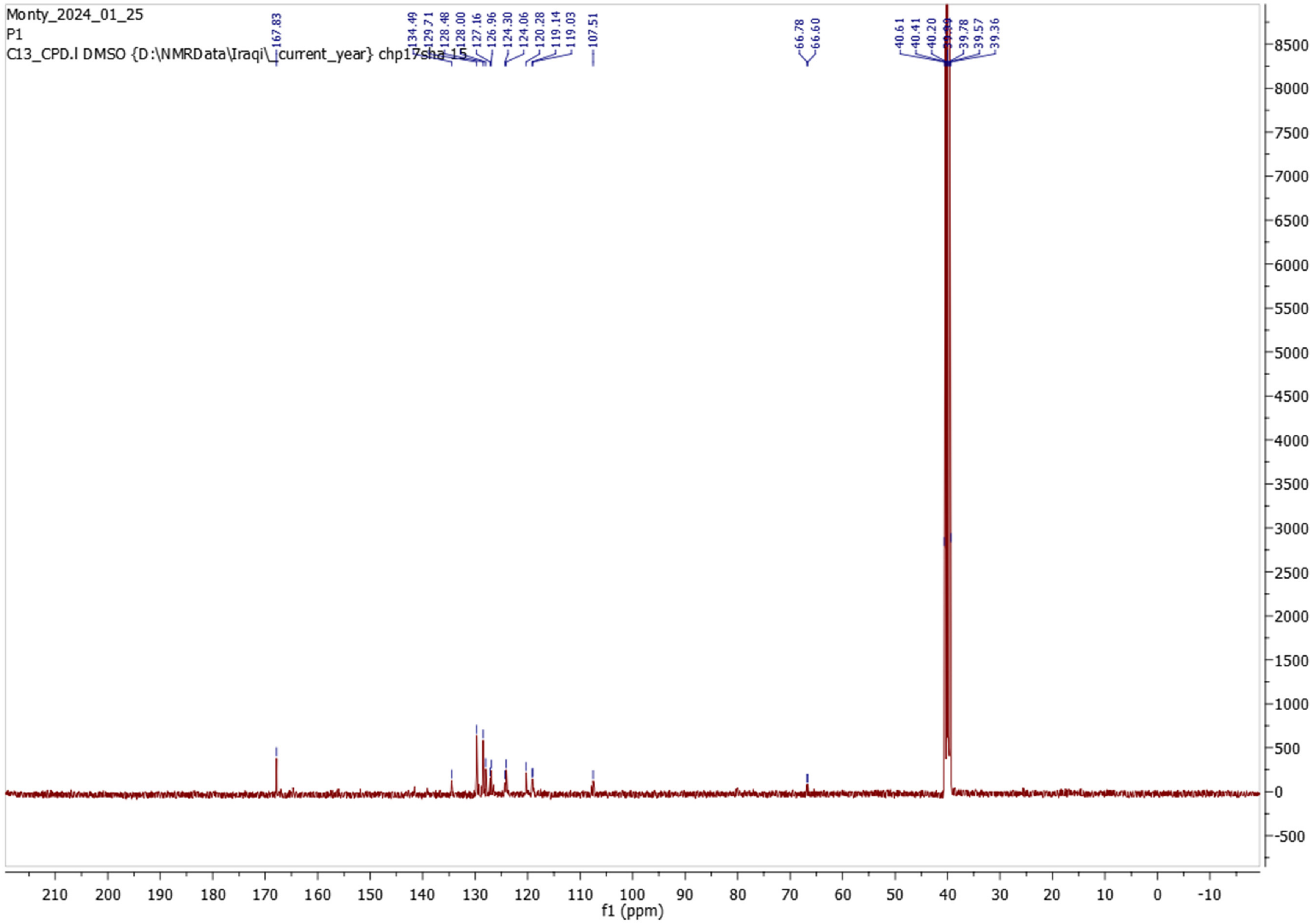

IR for 4-(2-cyano-3-(phenylamino)-3-(2-(2-(quinolin-4-yloxy)acetyl)hydrazineylidene) propanamido)benzamide **7b**

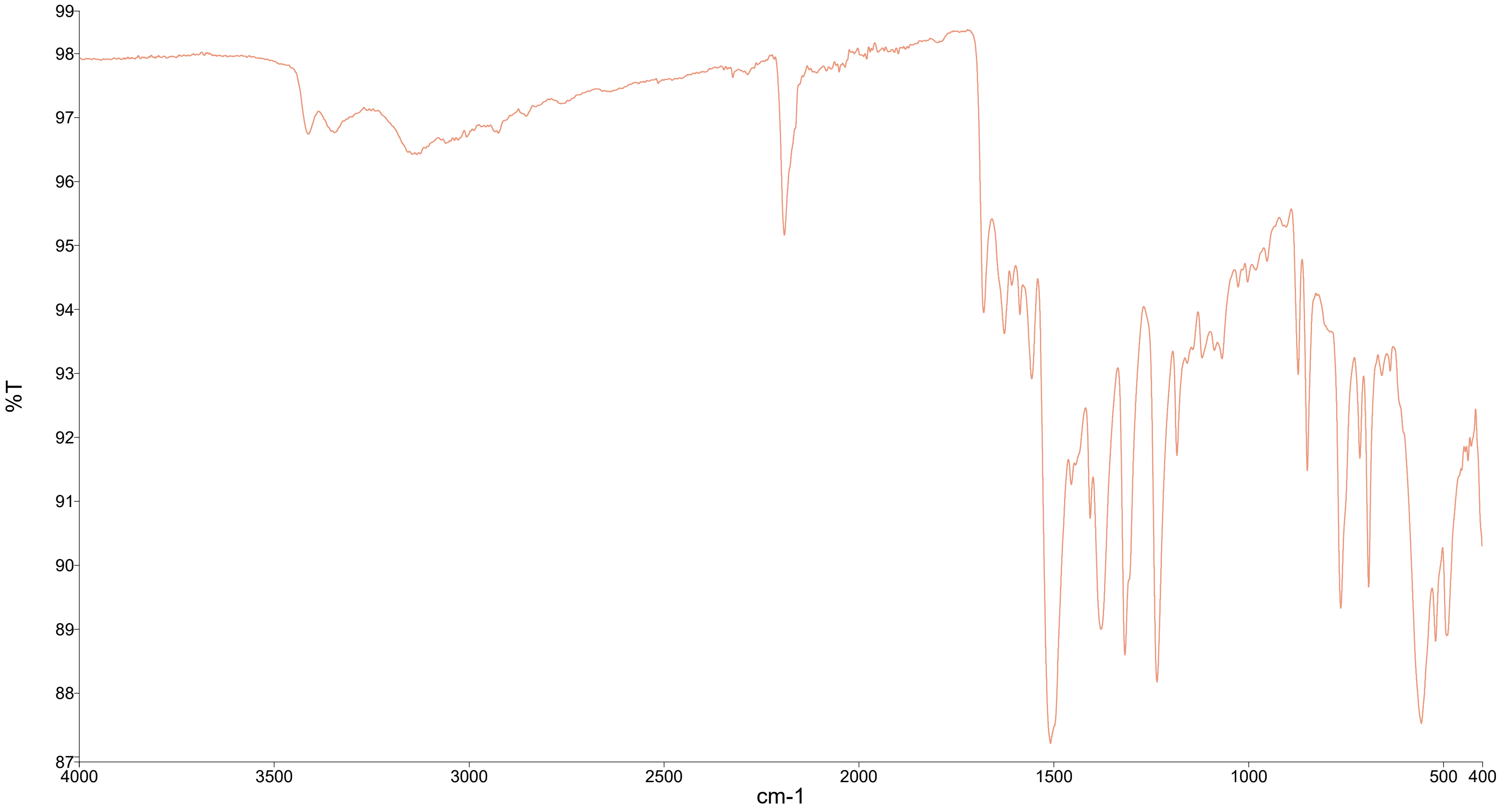

^{1}H NMR for 4-(2-cyano-3-(phenylamino)-3-(2-(2-(quinolin-4-yloxy)acetyl)hydrazineylidene) propanamido)benzamide **7b**

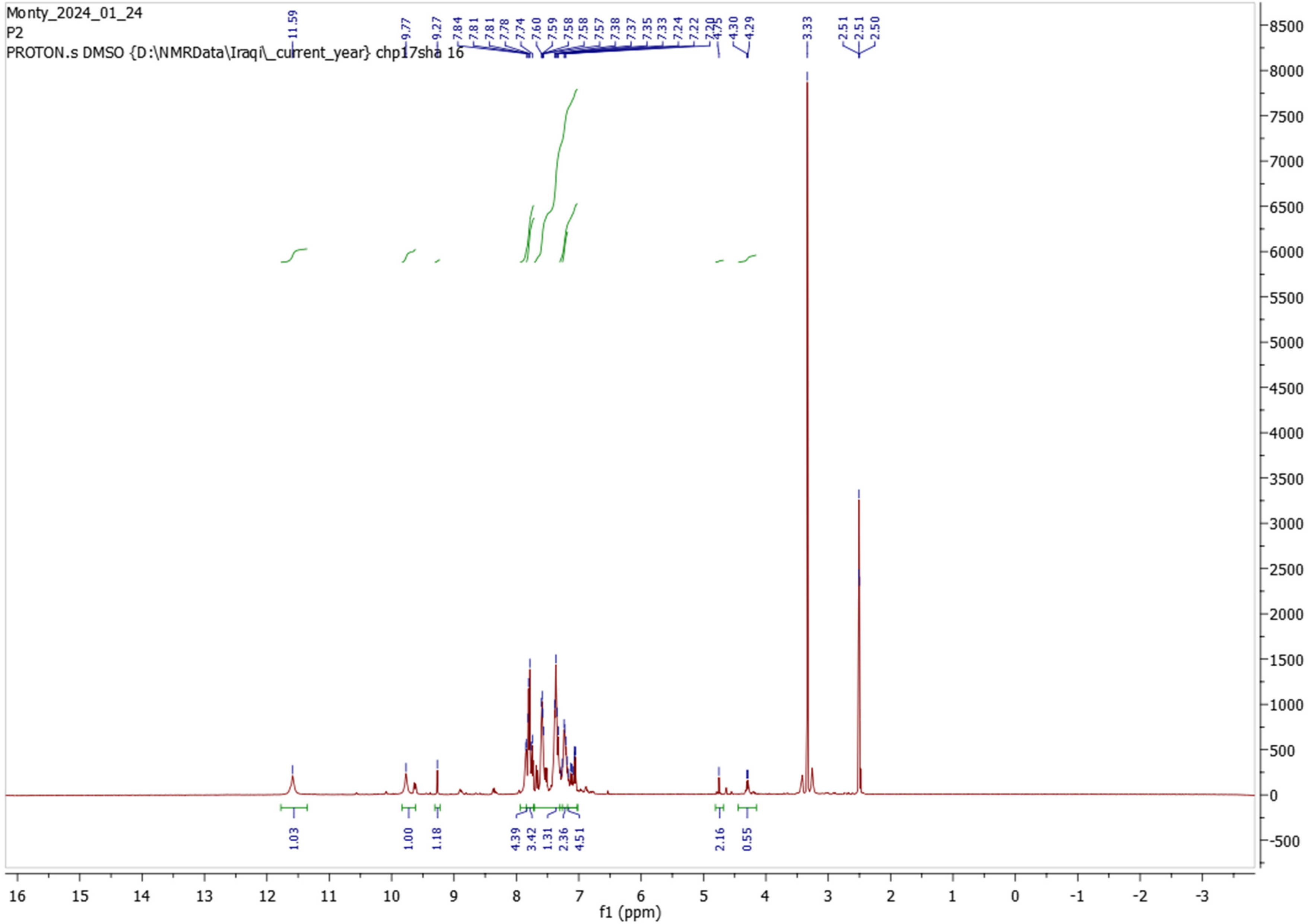

^{13}C NMR for 4-(2-cyano-3-(phenylamino)-3-(2-(2-(quinolin-4-yloxy)acetyl)hydrazineylidene) propanamido)benzamide **7b**

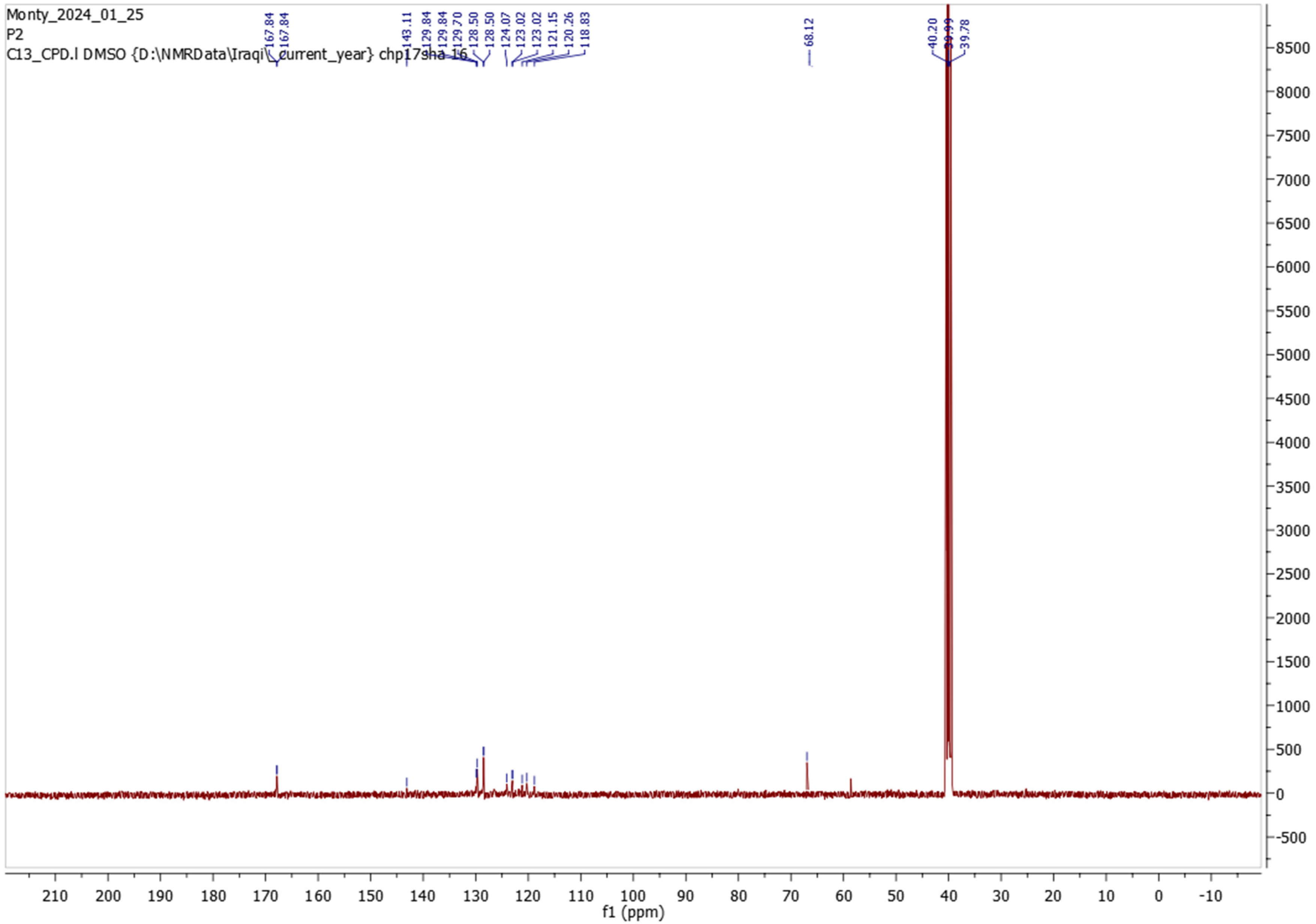

IR for 4-(2-cyano-3-(ethylamino)-3-(2-(2-(naphthalen-2-yloxy)acetyl)hydrazineylidene) propanamido)benzamide **7c**

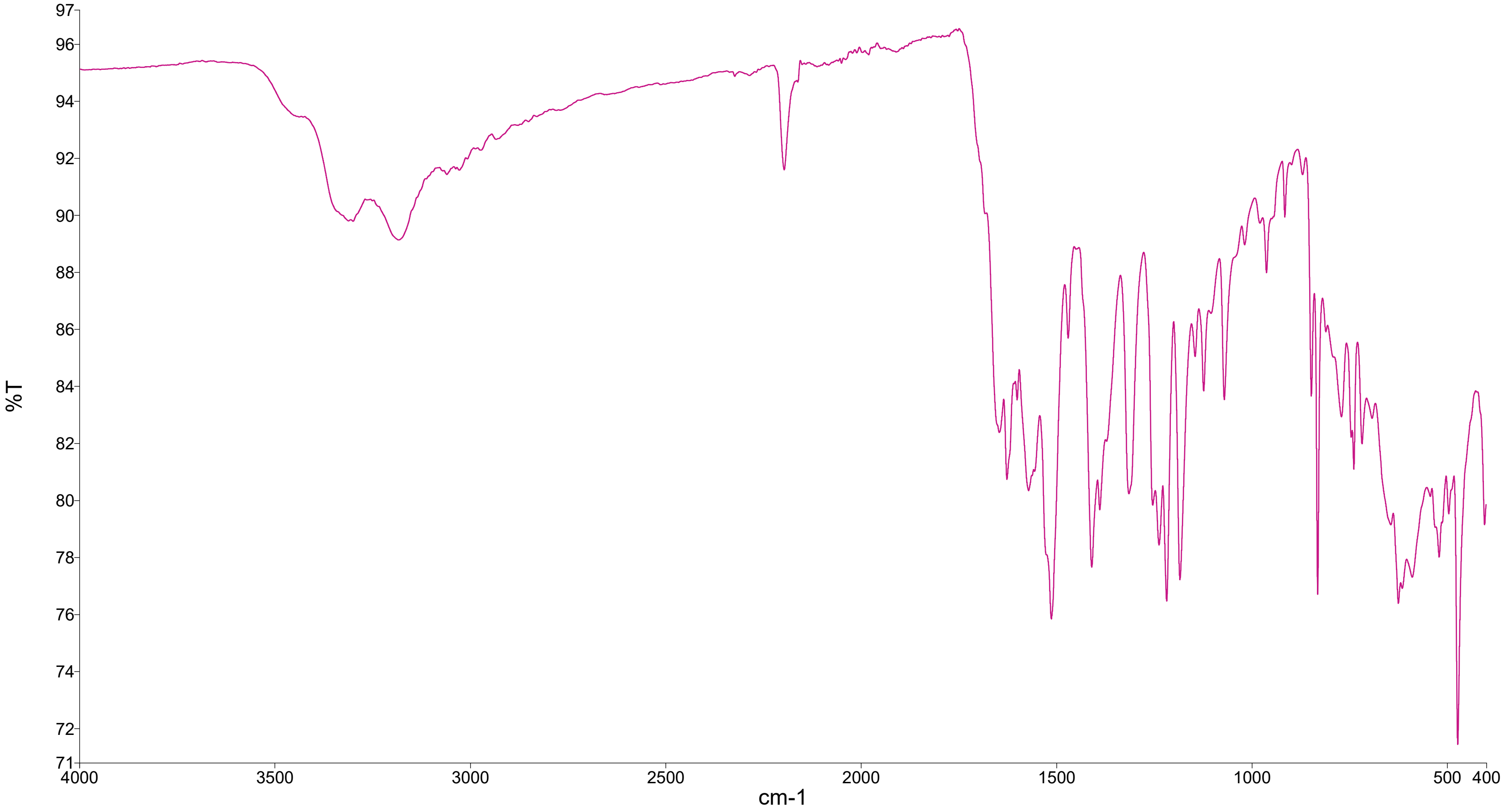

^{1}H NMR for 4-(2-cyano-3-(ethylamino)-3-(2-(2-(naphthalen-2-yloxy)acetyl)hydrazineylidene) propanamido)benzamide **7c**

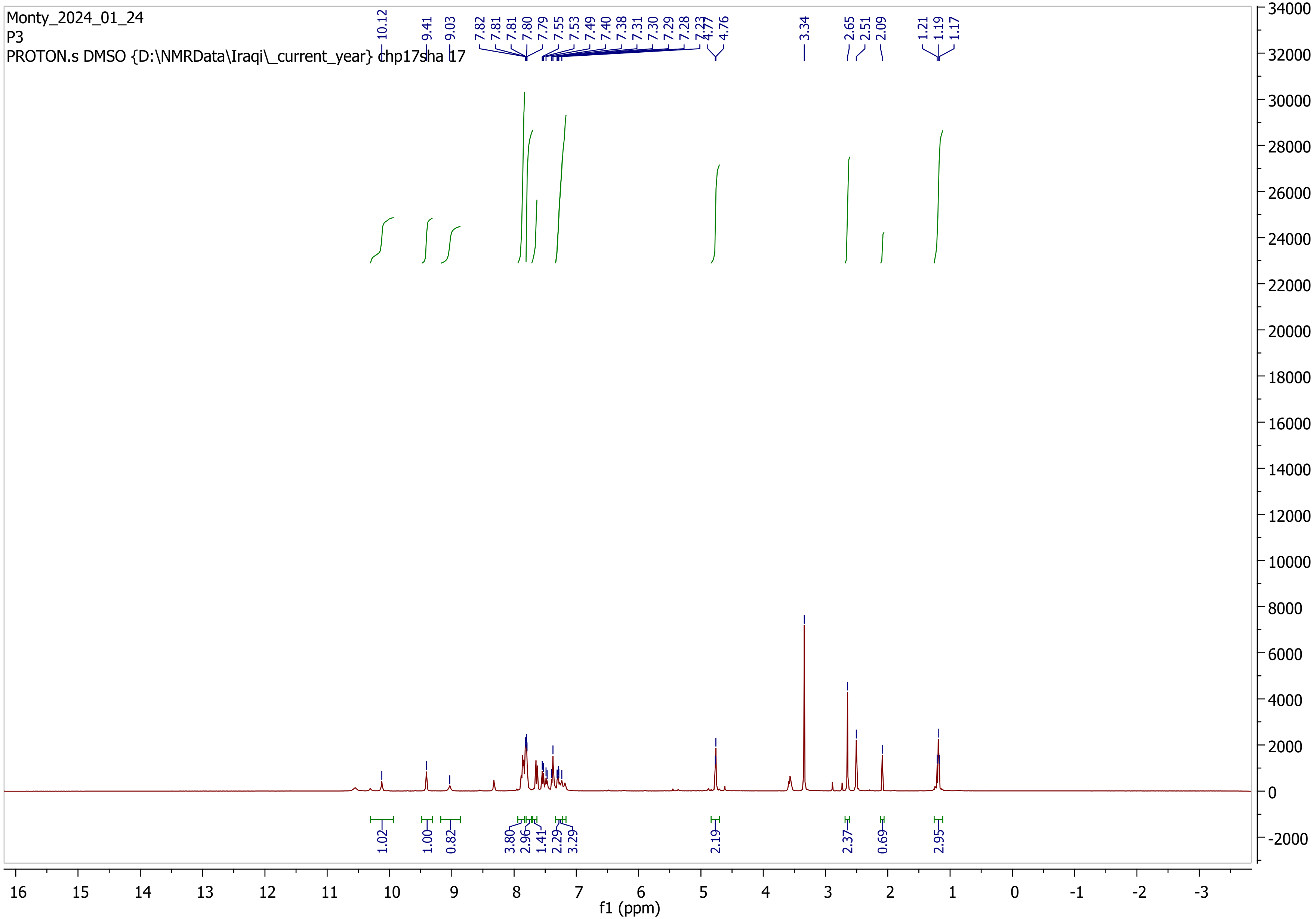

^{13}C NMR for 4-(2-cyano-3-(ethylamino)-3-(2-(2-(naphthalen-2-yloxy)acetyl)hydrazineylidene) propanamido)benzamide **7c**

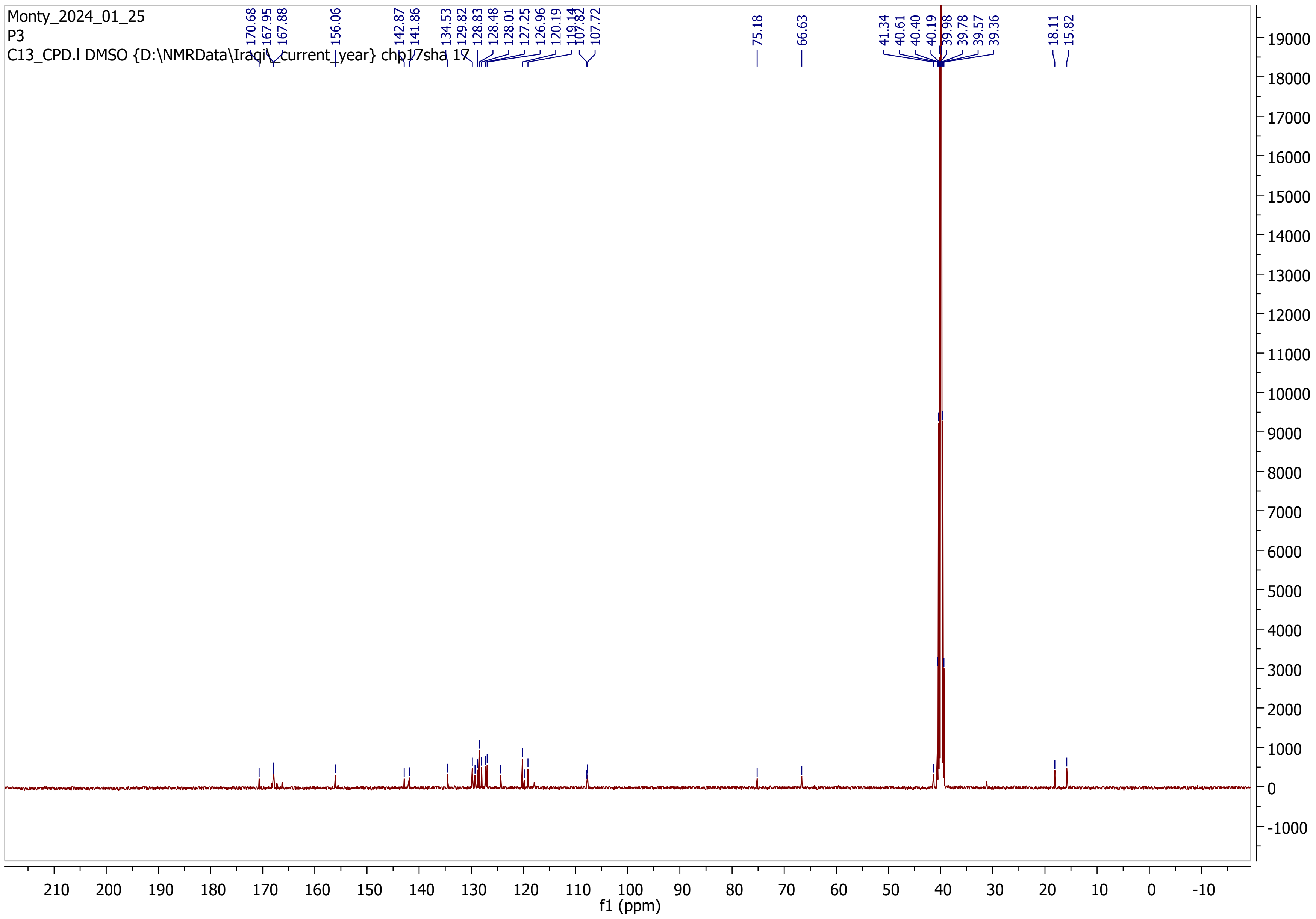

IR for *N*-(4-acetylphenyl)-2-cyano-3-(2-(2-(naphthalen-2-yloxy)acetyl)hydrazineylidene)-3-(phenylamino)propenamide **7d**

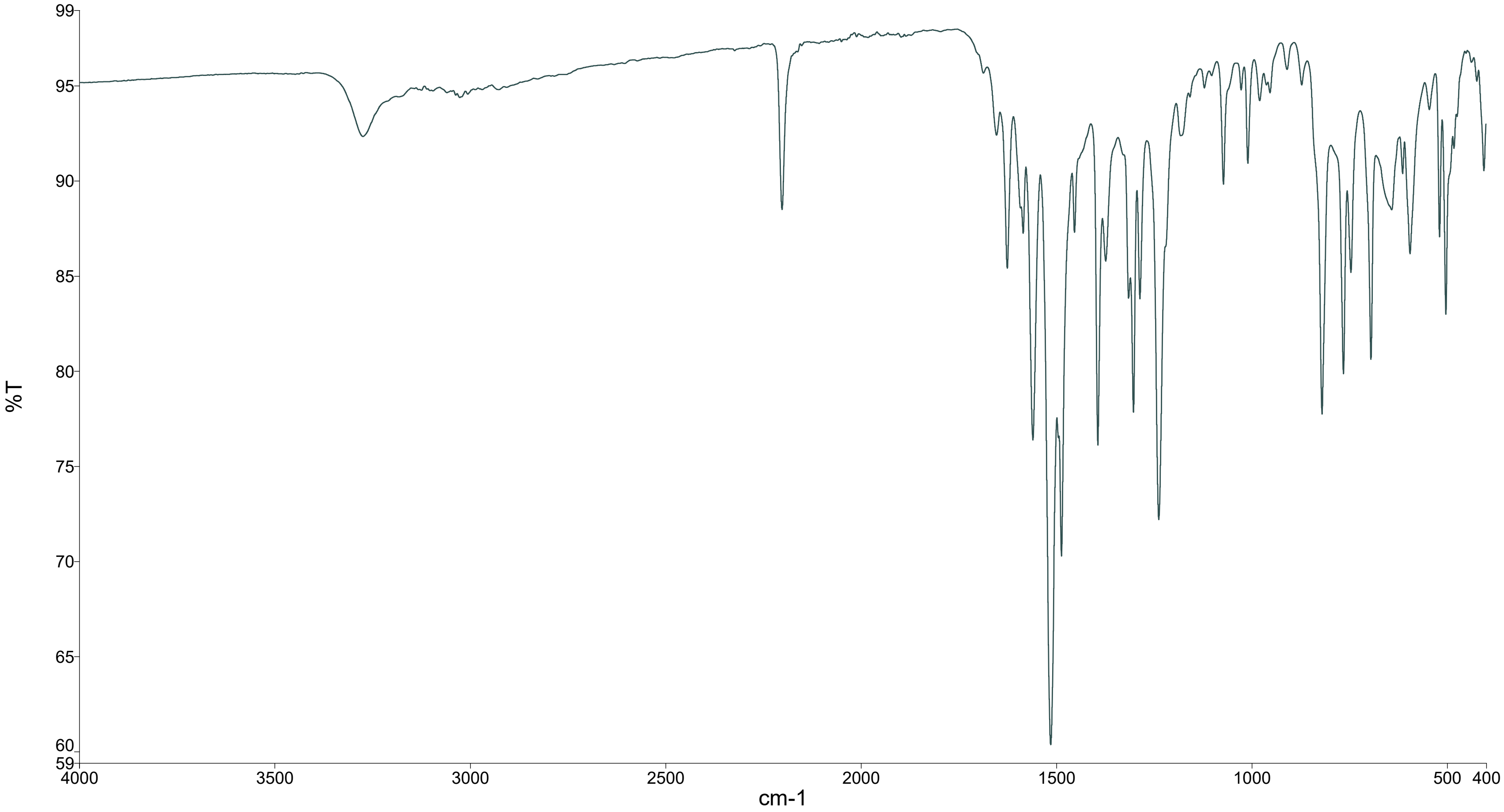

^{1}H NMR for *N*-(4-acetylphenyl)-2-cyano-3-(2-(2-(naphthalen-2-yloxy)acetyl)hydrazineylidene)-3-(phenylamino)propenamide **7d**

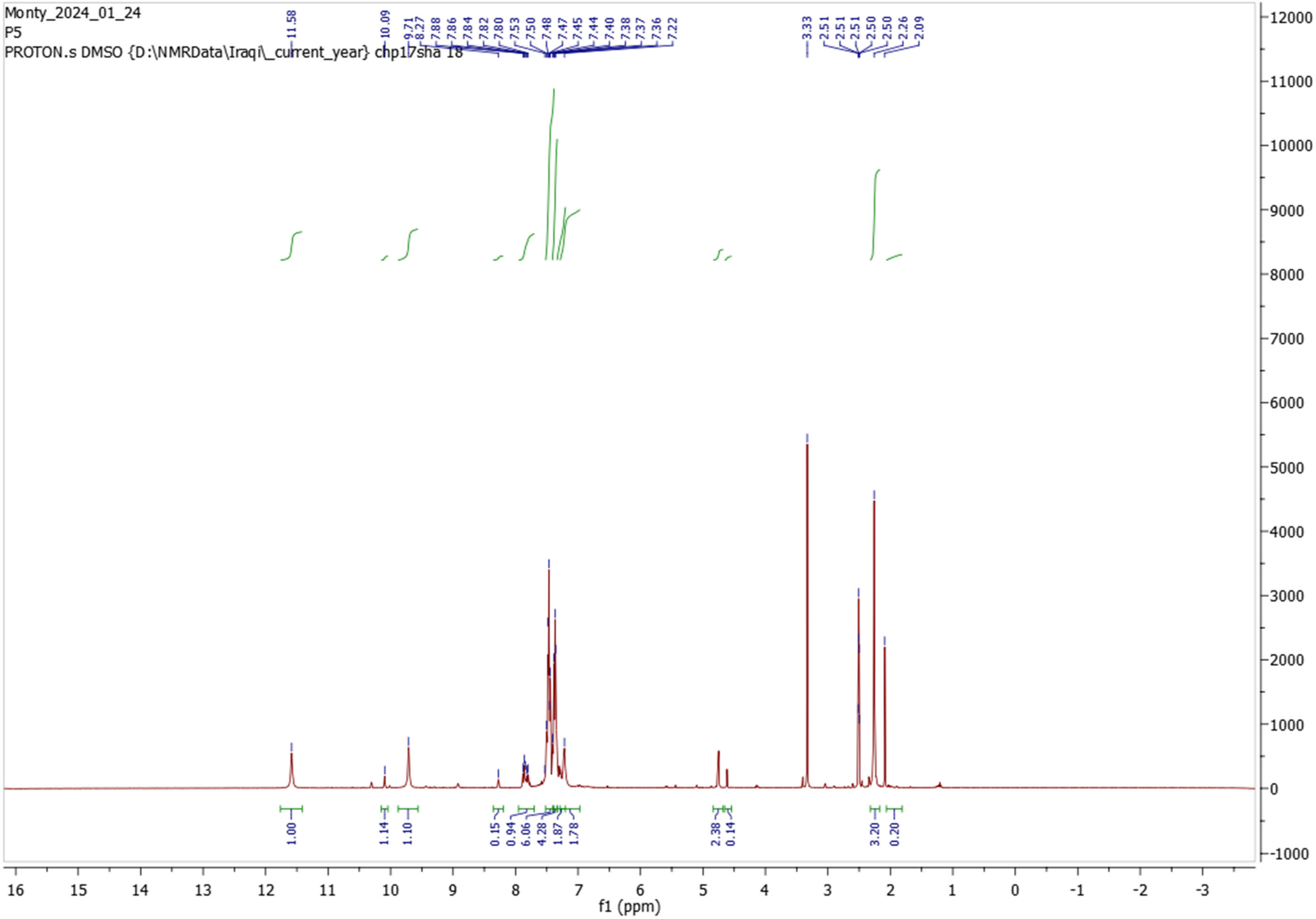

^{13}C NMR for *N*-(4-acetylphenyl)-2-cyano-3-(2-(2-(naphthalen-2-yloxy)acetyl)hydrazineylidene)-3-(phenylamino)propenamide **7d**

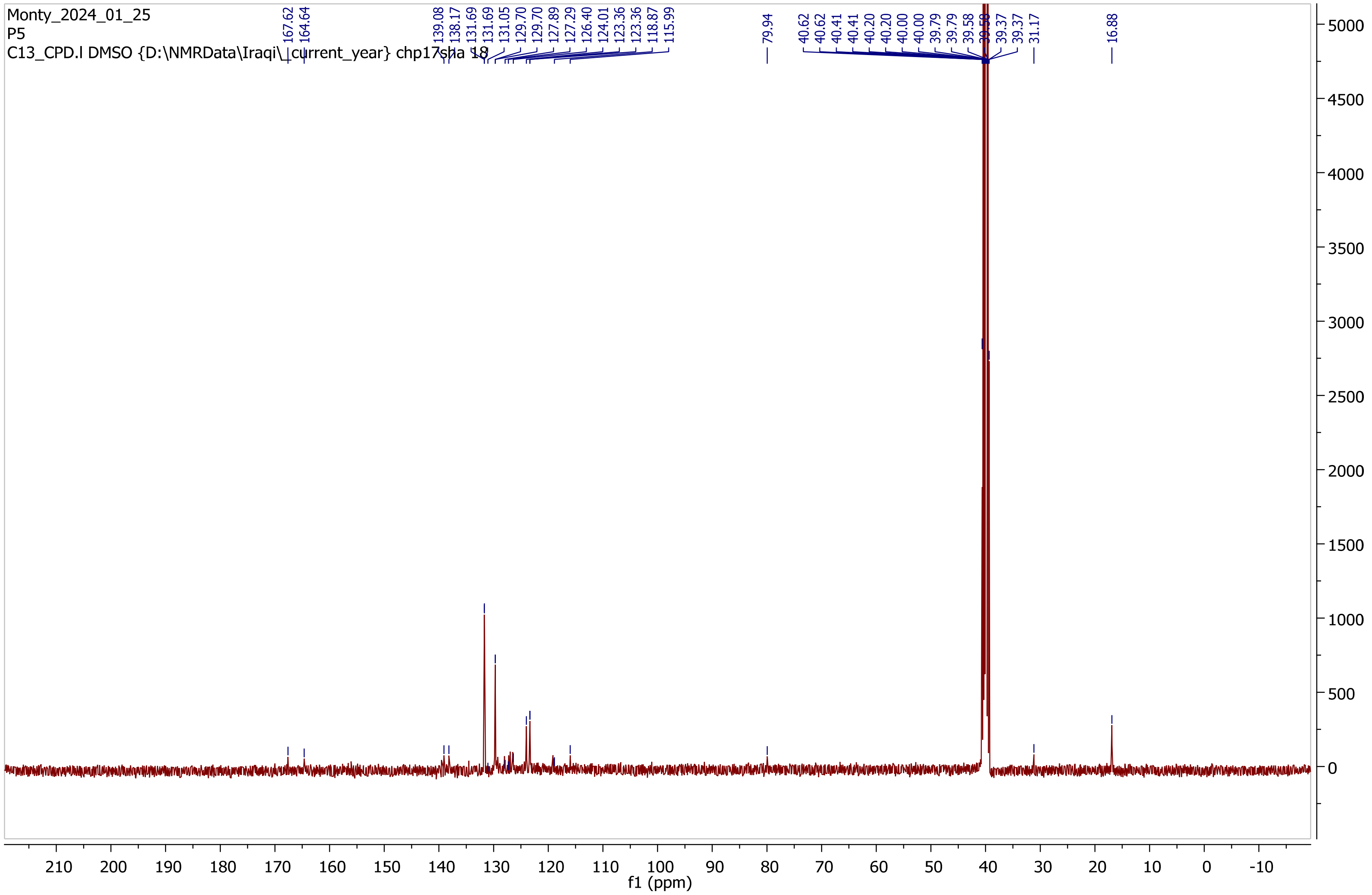

IR for *N*-(4-acetylphenyl)-2-cyano-3-(phenylamino)-3-(2-(2-(quinolin-4-yloxy)acetyl) hydrazineylidene)propenamide **7e**

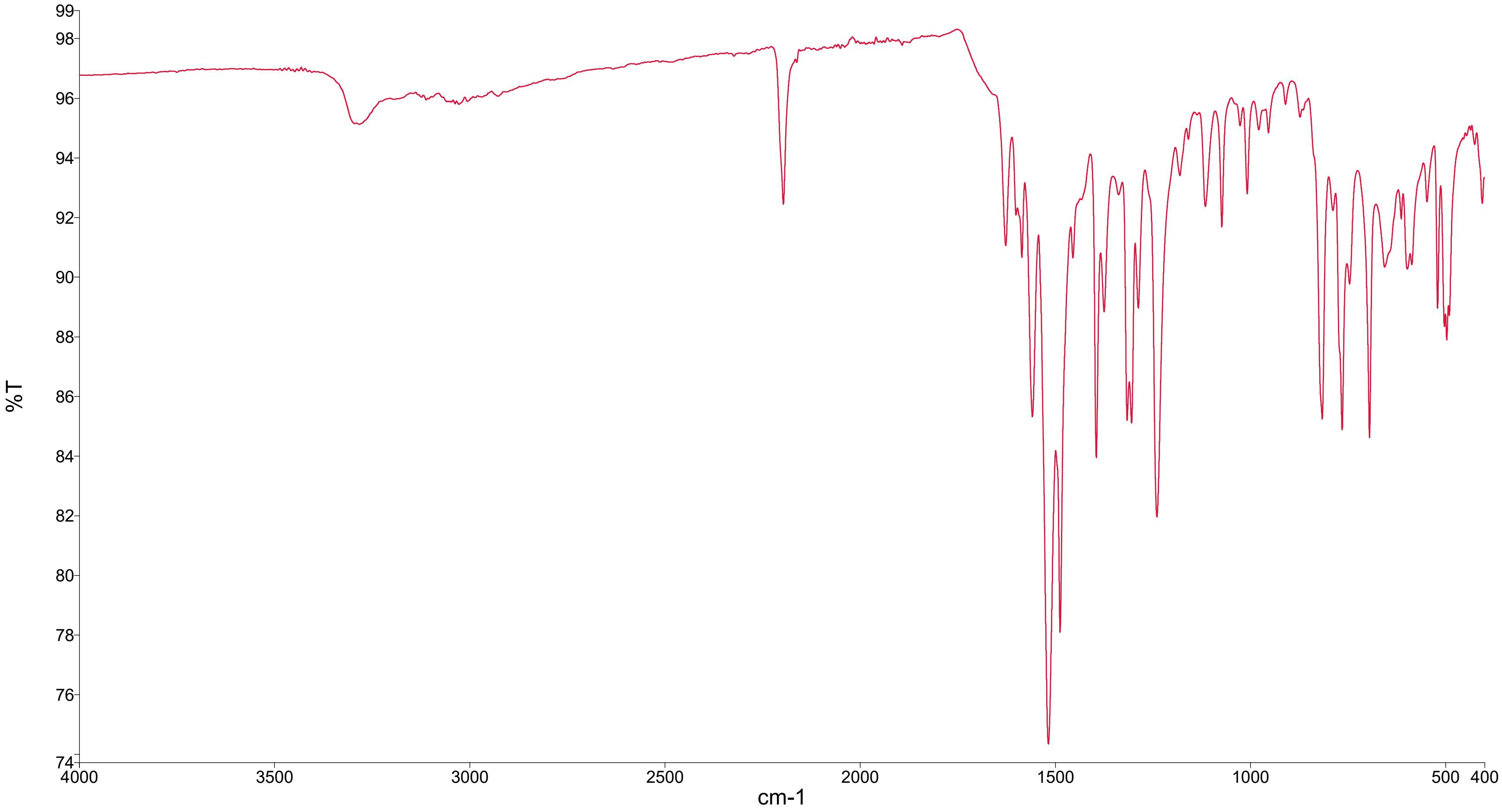

^{1}H NMR for *N*-(4-acetylphenyl)-2-cyano-3-(phenylamino)-3-(2-(2-(quinolin-4-yloxy)acetyl) hydrazineylidene)propenamide **7e**

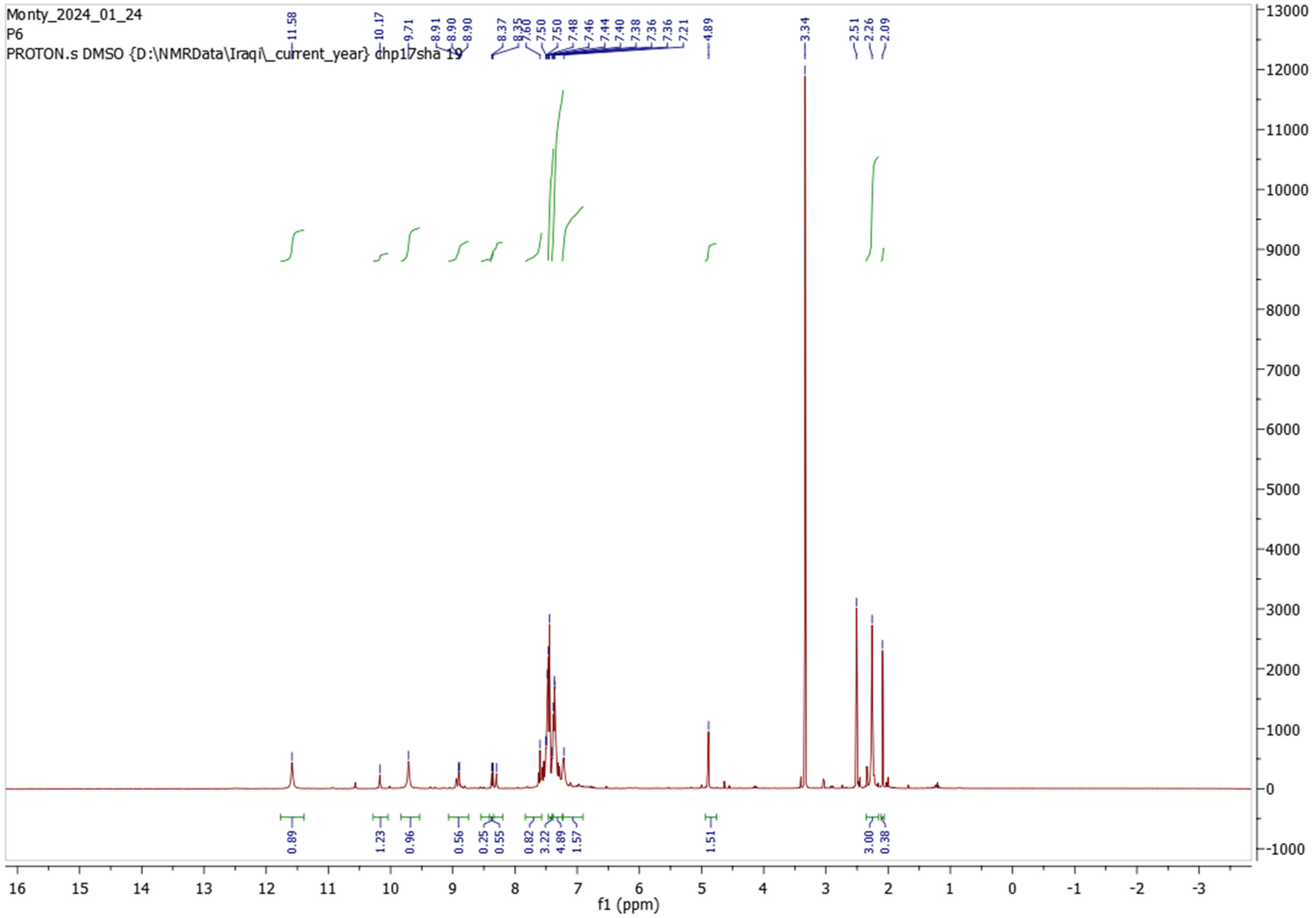

^{13}C NMR for *N*-(4-acetylphenyl)-2-cyano-3-(phenylamino)-3-(2-(2-(quinolin-4-yloxy)acetyl) hydrazineylidene)propenamide **7e**

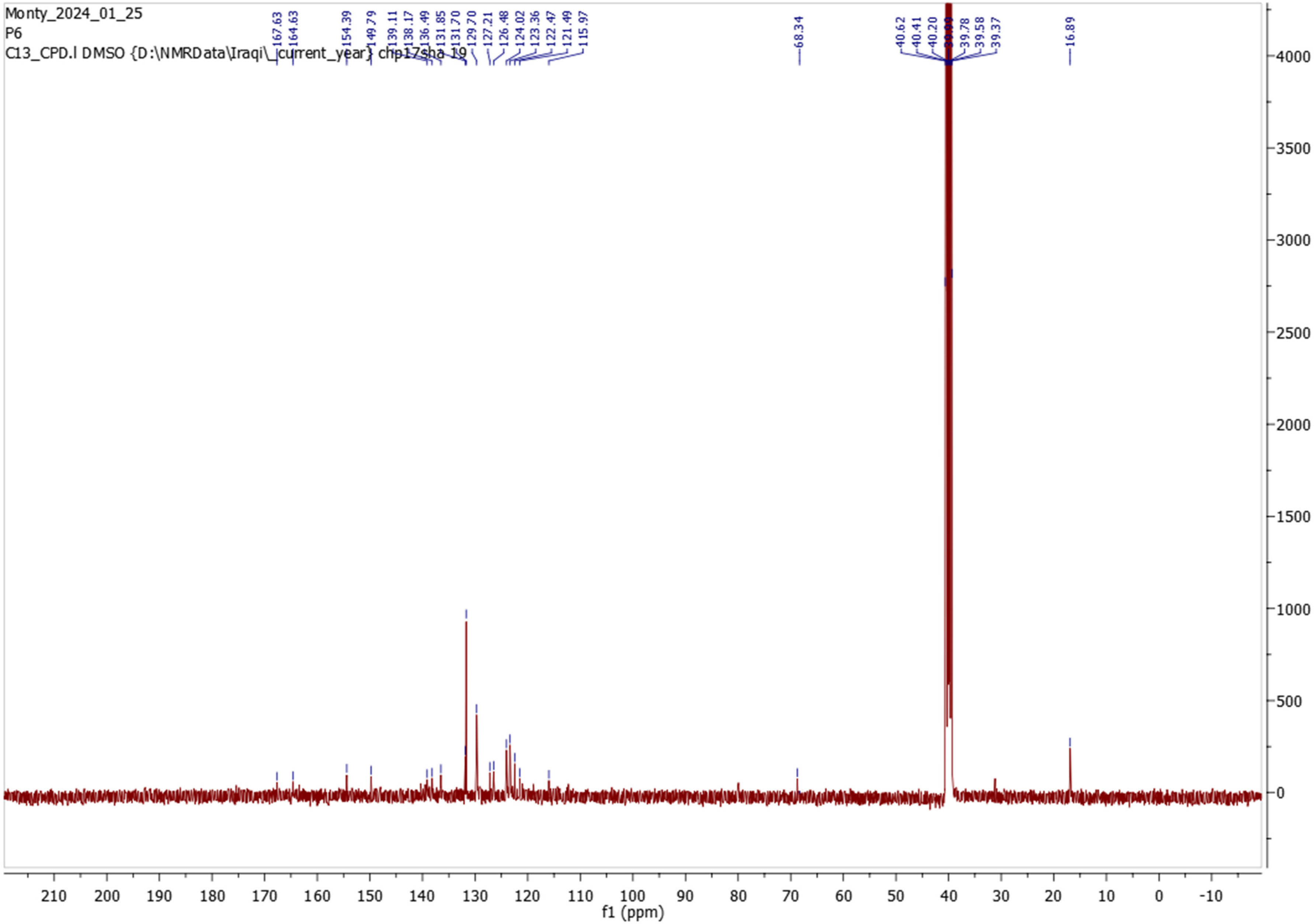

IR for *N*-(4-acetylphenyl)-2-cyano-3-(ethylamino)-3-(2-(2-(naphthalen-2-yloxy)acetyl) hydrazineylidene)propenamide **7f**

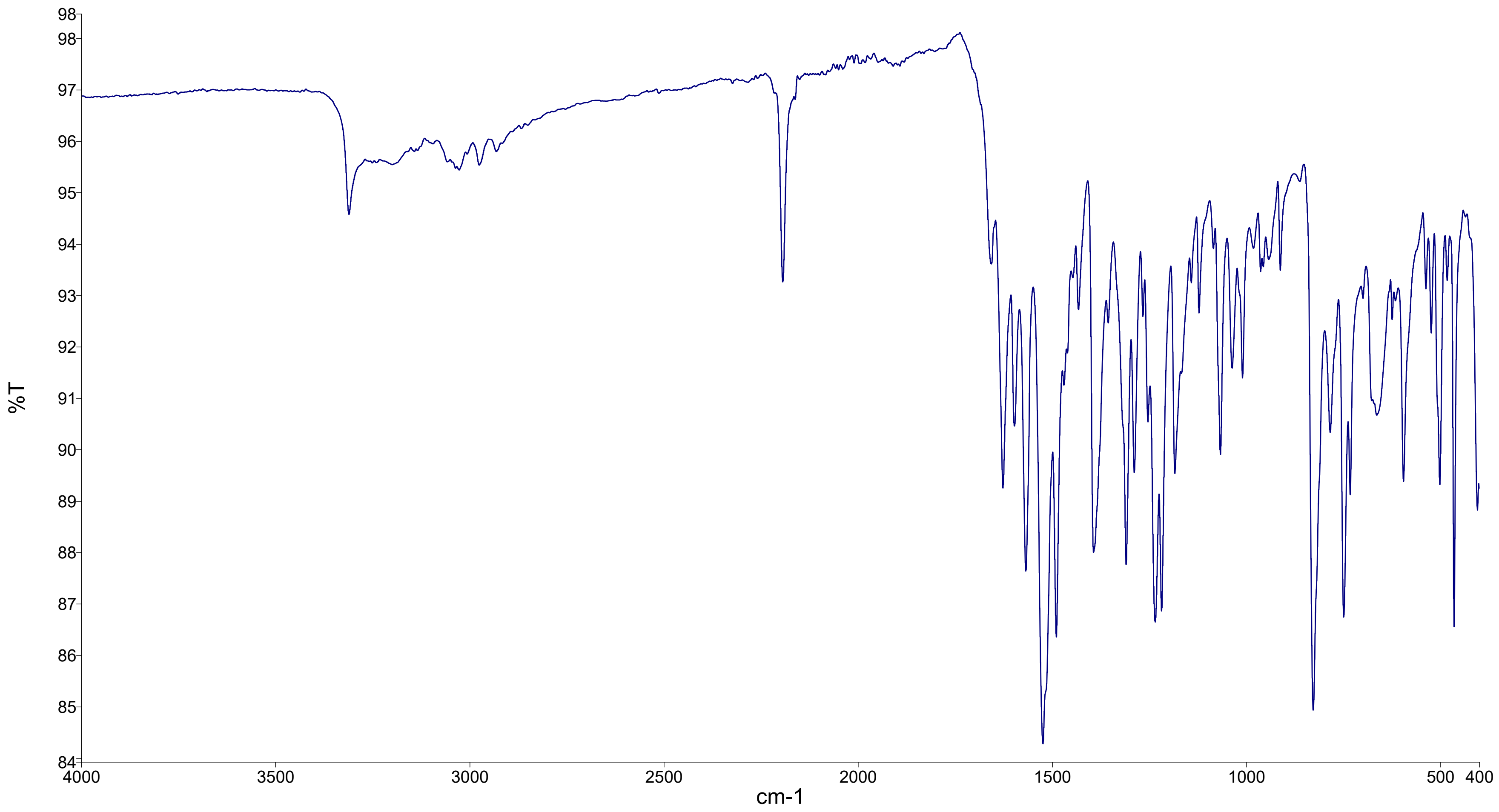

^{1}H NMR for *N*-(4-acetylphenyl)-2-cyano-3-(ethylamino)-3-(2-(2-(naphthalen-2-yloxy)acetyl) hydrazineylidene)propenamide **7f**

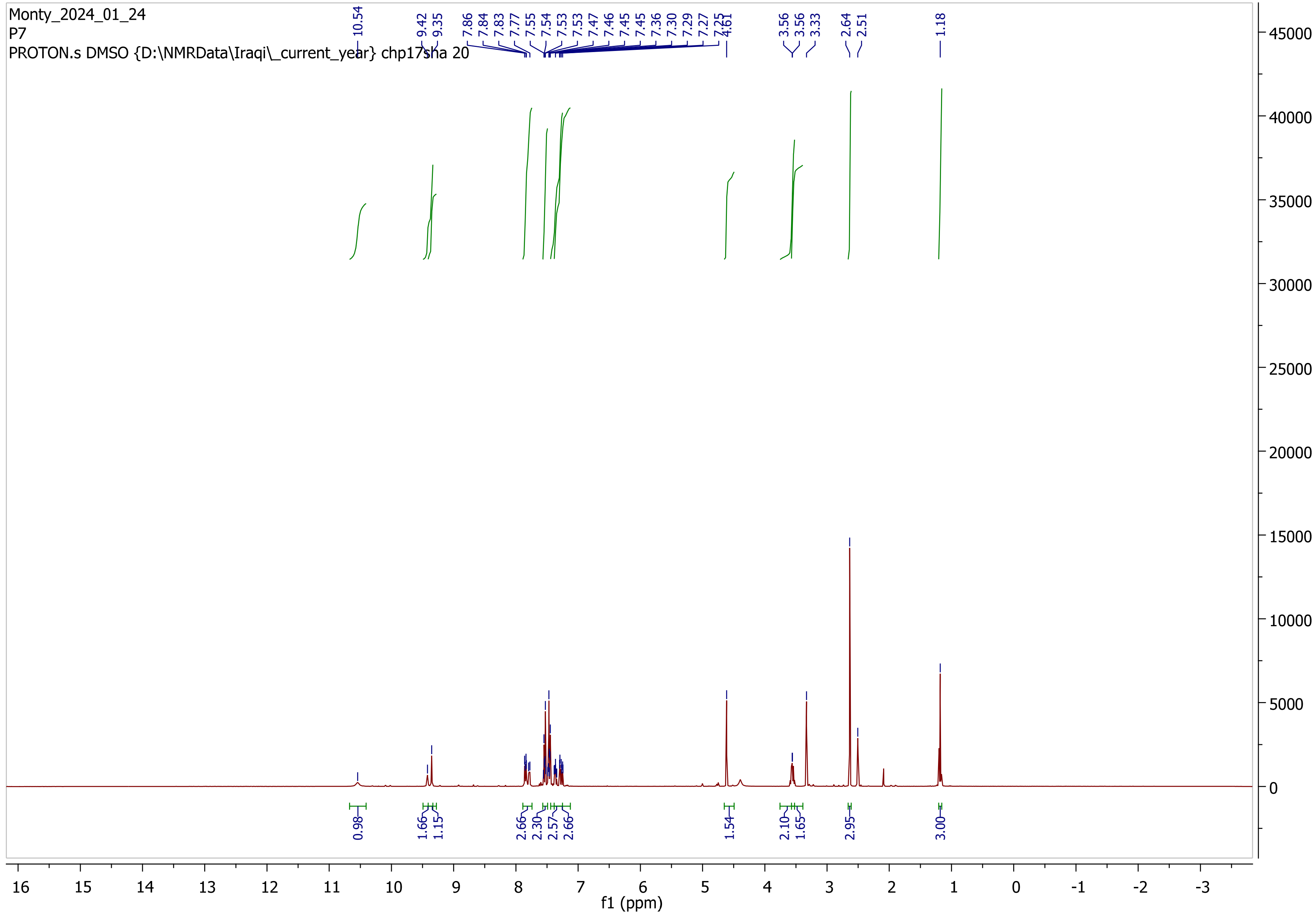

^{13}C NMR for *N*-(4-acetylphenyl)-2-cyano-3-(ethylamino)-3-(2-(2-(naphthalen-2-yloxy)acetyl) hydrazineylidene)propenamide **7f**

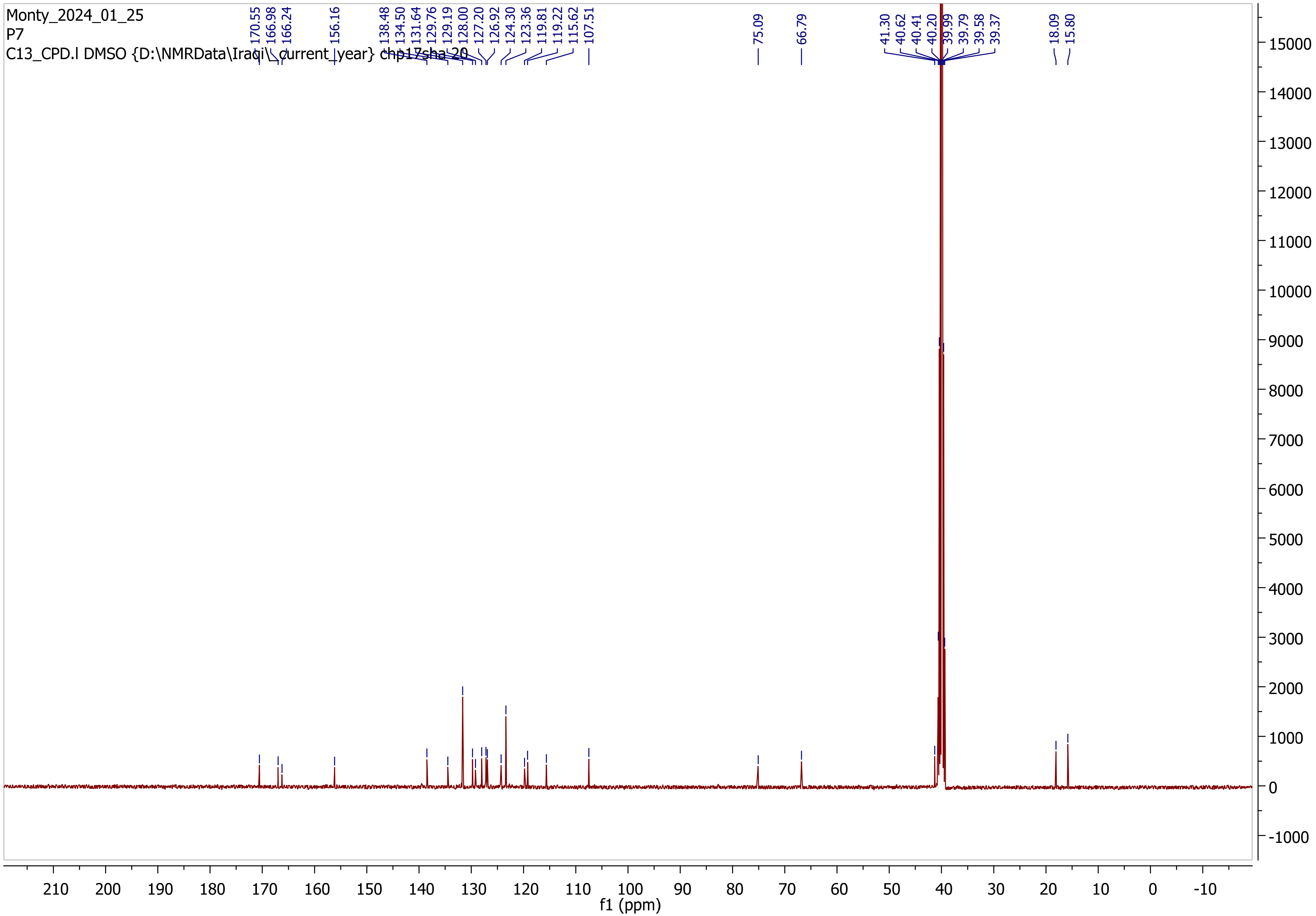

IR for *N*-(4-acetylphenyl)-2-cyano-3-(ethylamino)-3-(2-(2-(quinolin-4-yloxy)acetyl) hydrazineylidene)propenamide **7g**

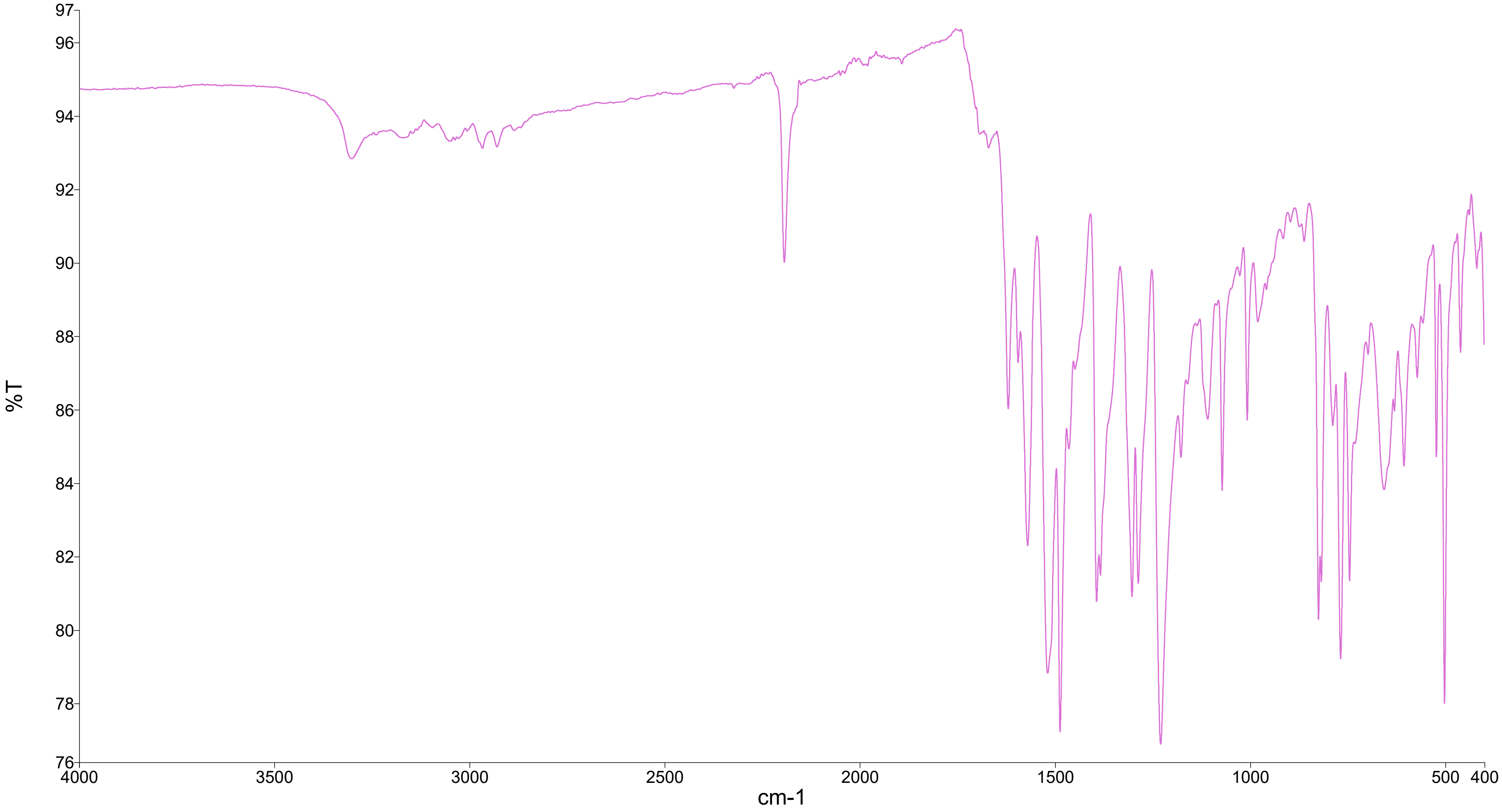

^{1}H NMR for *N*-(4-acetylphenyl)-2-cyano-3-(ethylamino)-3-(2-(2-(quinolin-4-yloxy)acetyl) hydrazineylidene)propenamide **7g**

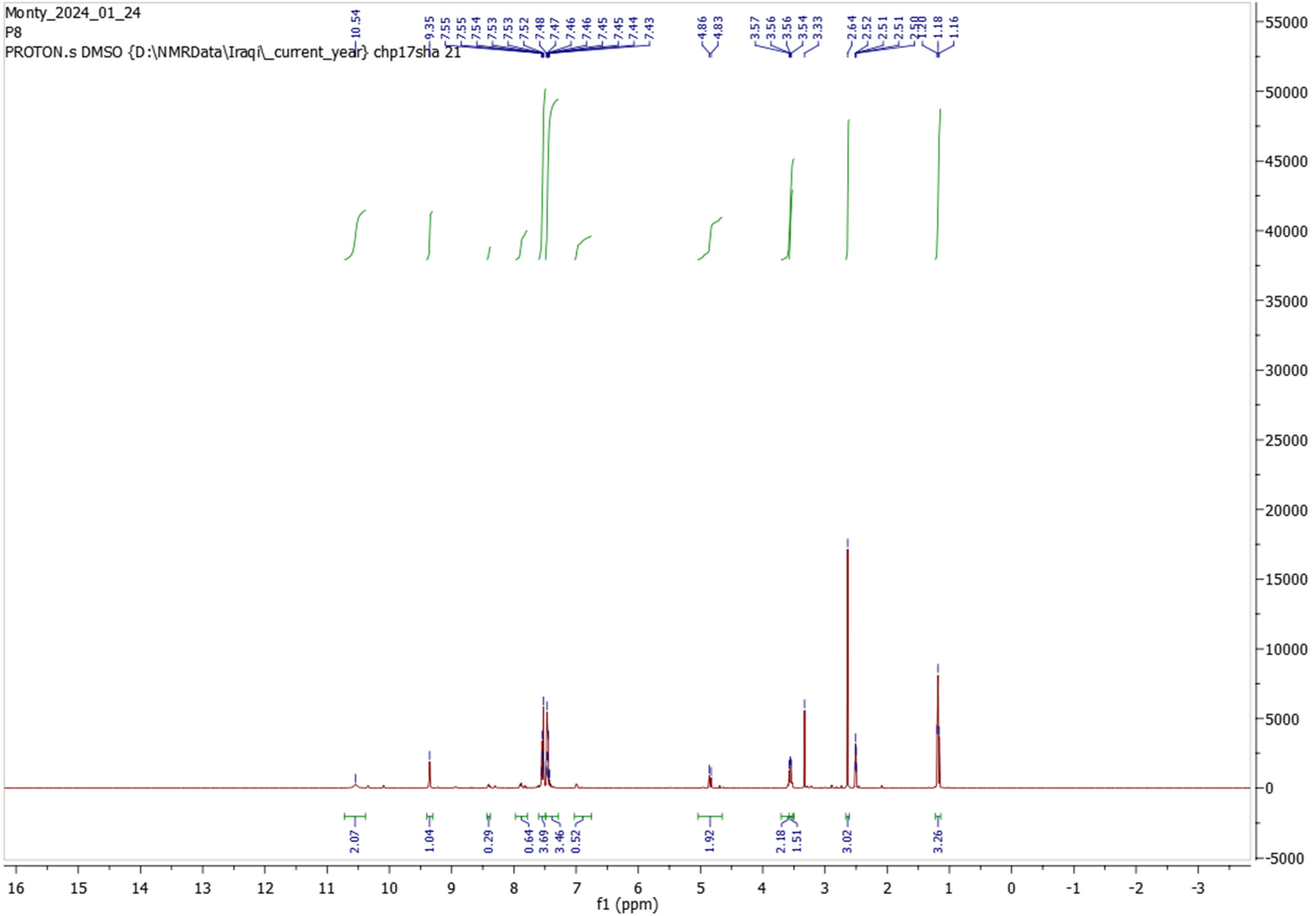

^{13}C NMR for *N*-(4-acetylphenyl)-2-cyano-3-(ethylamino)-3-(2-(2-(quinolin-4-yloxy)acetyl) hydrazineylidene)propenamide **7g**

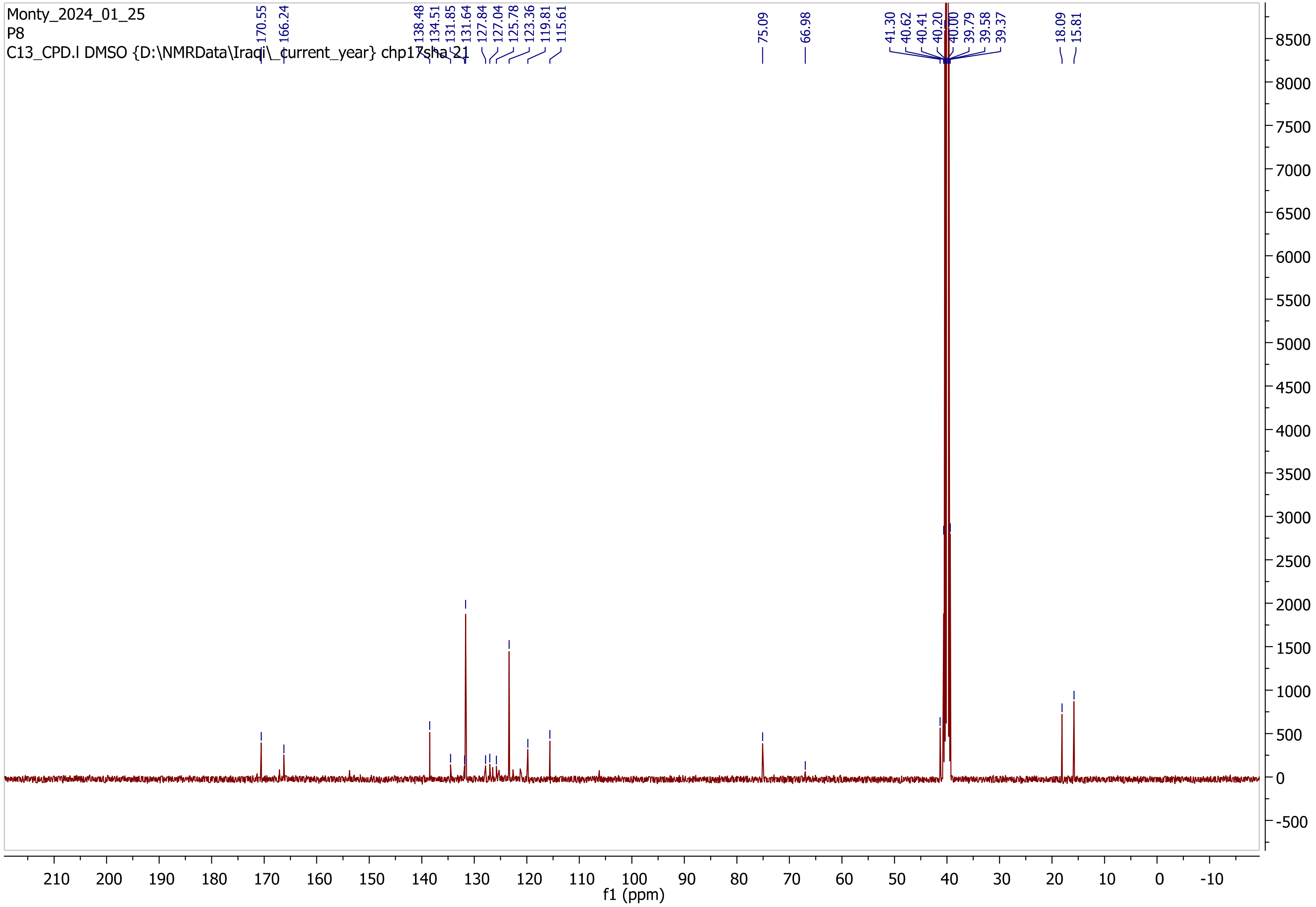

Supplement: Supplementary file 1 [file pharmaceuticals-17-01692-s001.zip › pharmaceuticals-3304118-supplementary.pdf]
